# Two‐Photon Fluorescence and Second‐Harmonic Generation Microscopy: Principles and Biomedical Applications

**DOI:** 10.1002/jbio.70369

**Published:** 2026-09-29

**Authors:** Kausalya Neelavara Makkithaya, Gagan Raju, Subir Das, Ajeetkumar Patil, Rajesh Kumar, Yury V. Kistenev, Guan‐Yu Zhuo, Nirmal Mazumder

**Affiliations:** ^1^ Department of Biophysics, Manipal School of Life Sciences Manipal Academy of Higher Education Manipal India; ^2^ Department of Chemistry University of Zurich Zurich Switzerland; ^3^ Manipal Institute of Applied Physics Manipal Academy of Higher Education Manipal India; ^4^ Department of Biomedical Engineering Indian Institute of Technology Ropar India; ^5^ Laboratory of Biophotonics Tomsk State University Tomsk Russia; ^6^ Institute of Biophotonics National Yang Ming Chiao Tung University Taipei Taiwan

**Keywords:** biomedical research, fluorescence lifetime imaging, nonlinear optical microscopy, second‐harmonic generation microscopy, two‐photon microscopy

## Abstract

Two‐photon microscopy (TPM) has revolutionized biological imaging by enabling high‐resolution, deep‐tissue visualization with reduced photobleaching and photodamage. This review discusses complementary nonlinear optical imaging modalities such as two‐photon fluorescence (TPF), second‐harmonic generation (SHG), and fluorescence lifetime imaging (FLIM) microscopy that together provide structural, molecular, and metabolic information. The review begins by introducing the basic principles, instrumentation, and capabilities of two‐photon fluorescence microscopy and FLIM. Followed by the development of SHG microscopy, highlighting advances from conventional SHG imaging to polarization‐resolved, circular dichroism, and Stokes–Mueller polarimetric approaches. The biomedical applications of these complementary techniques are subsequently discussed, followed by an overview of multimodal imaging strategies that combine structural, molecular, and functional information. Collectively, these modalities represent a versatile and rapidly evolving imaging toolkit with broad potential to advance biomedical research, disease diagnosis, and precision medicine.

## Graphical abstract

- Compared with single‐photon methods, TPM enables deep‐tissue imaging using near‐infrared light, with excitation confined to the focal plane, thereby reducing photodamage.
- SHG microscopy images noncentrosymmetric biological structures such as collagen and myosin without fluorescent labels, offering direct structural contrast.

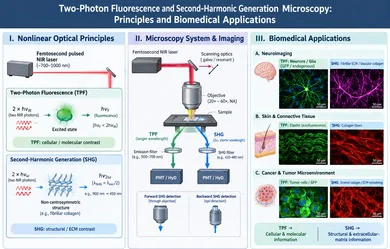

## 1 Introduction

Nonlinear optical microscopy has substantially changed the way researchers image biological samples. The foundations of the field rest on two developments: the theoretical prediction of two‐photon absorption by Maria Göppert‐Mayer in the 1930s, which showed that two photons of lower energy could jointly excite a fluorophore when absorbed simultaneously [1], and the subsequent availability of pulsed ruby lasers in the 1960s, which supplied the photon densities needed to observe multiphoton processes [2, 3, 4]. Practical two‐photon microscopy (TPM) became possible in the 1990s by Winfried Denk, James Strickler, and Watt Webb, who demonstrated that mode‐locked femtosecond lasers could deliver the high peak intensities required for efficient two‐photon absorption while keeping the average power low enough to avoid tissue damage [5]. This demonstration opened up deep‐tissue, live‐sample imaging in ways that single‐photon confocal microscopy could not match. Compared with confocal and wide‐field fluorescence microscopy, nonlinear techniques offer several practical advantages [6]. Because two‐photon absorption is a quadratic process, excitation occurs only at the tight focus of the laser beam. The out‐of‐focus regions receive no significant illumination, so photobleaching and photodamage are largely confined to the focal volume rather than distributed throughout the sample depth [7]. This matters for longitudinal studies where the same tissue must be imaged repeatedly. Near‐infrared (NIR) excitation wavelengths (typically 700–1100 nm) are scattered less in biological tissue than the shorter visible wavelengths used in traditional optical imaging methods, permitting imaging hundreds of micrometers below the tissue surface [8]. The nonlinear absorption process also provides inherent optical sectioning without a pinhole, yielding images with a low out‐of‐focus background, which improves contrast in thick, three‐dimensional samples. Furthermore, a single NIR excitation wavelength can simultaneously excite multiple fluorophores with different emission spectra, supporting multiplexed imaging of several molecular targets within the same sample [9].

Among the nonlinear optical microscopy techniques comprising third‐harmonic generation (THG) and coherent anti‐Stokes Raman scattering (CARS) microscopy, TPM and second‐harmonic generation (SHG) microscopy have seen the widest biomedical adoption [10, 11, 12]. TPM is well suited for imaging thick samples in neuroscience, developmental biology, and live‐cell studies [10, 13]. SHG microscopy generates contrast exclusively from noncentrosymmetric molecular assemblies such as fibrillar collagen and myosin, providing label‐free images that reflect the structural organization of connective tissues and muscle [11]. CARS microscopy generates vibrational contrast by exploiting the interaction of a pump and a Stokes laser to produce a coherent anti‐Stokes signal, enabling high‐speed imaging of lipid distributions and other chemical species without labels [12]. This review covers the principles and applications of TPM and SHG microscopy, describes the instrumentation used for each, and discusses the biomedical application via structural and functional information from the images they produce.

### 1.1 Principle of Two‐Photon Excitation (TPE)

In TPE, a fluorophore absorbs the energy of two photons simultaneously, each carrying approximately half the energy needed to reach the excited state. Because this is a second‐order process, the probability of excitation scales with the square of the instantaneous light intensity rather than linearly with it [14]. Consequently, two‐photon absorption is largely confined to the focal volume of the objective lens, where the photon density is maximal, whereas regions above and below the focal plane receive insufficient photon flux to induce significant excitation (Figure 1) [15]. This intrinsic localization of fluorescence generation results in optical sectioning capability and eliminates the need for a confocal pinhole. The confined excitation geometry provides several practical benefits. NIR wavelengths used for TPE scatter less in tissue than the visible light used in conventional fluorescence microscopy, allowing imaging at greater sample depths [16]. As only fluorophores at the focal point are excited, out‐of‐focus structures are not bleached or damaged during image acquisition [17]. The quadratic intensity dependence also improves image contrast relative to single‐photon methods, and compatibility with fluorescence lifetime imaging microscopy (FLIM) adds a quantitative dimension by reporting on the molecular environment of each fluorophore [18, 19].

**FIGURE 1:**
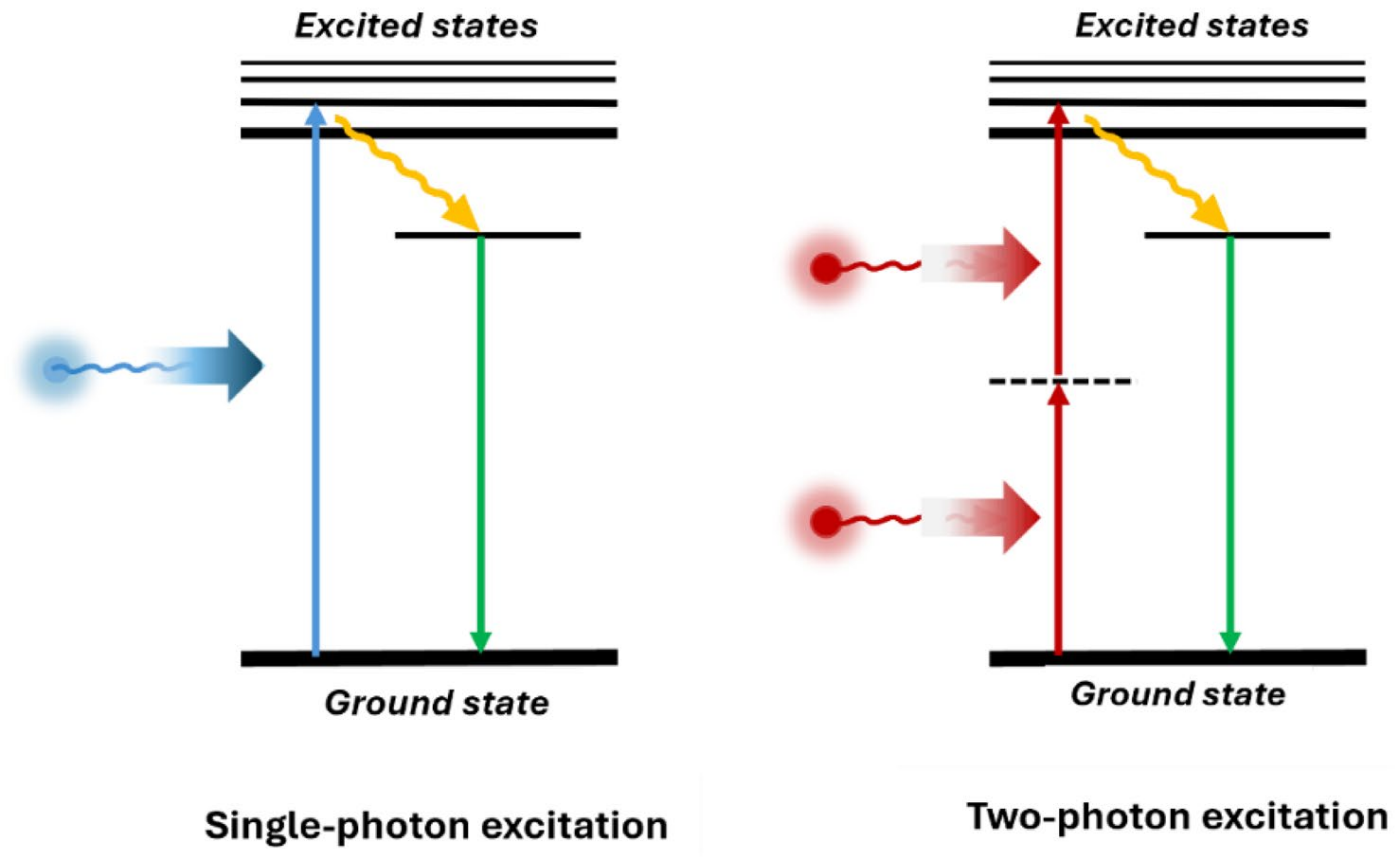
Schematic of energy transfer in single‐photon (left) and two‐photon (right) excitation processes. In TPE, two lower‐energy photons are absorbed simultaneously to populate the same excited state reached by one higher‐energy photon in conventional fluorescence.

## 2 Two‐Photon Fluorescence (TPF) Microscopy

Following its introduction in 1990 [20], TPM has become a broadly used tool in biomedical research, with neuroscience, developmental biology, and cancer biology among its primary application areas. The technique relies on TPE: two photons must arrive within approximately 1 fs of each other to jointly drive the fluorophore from its ground state to the excited state from which emission occurs. The resulting fluorescence intensity is proportional to the square of the excitation power. Spatial confinement of this excitation to the focal volume gives a TPM spatial resolution comparable to that of confocal microscopy without the pinhole, and the NIR excitation used allows imaging of structures well below the tissue surface. Advances in pulsed laser technology, detector sensitivity, and scanning optics have steadily extended these capabilities since the initial demonstration [6, 21].

### 2.1 TPE: Process and Mechanism

In one‐photon fluorescence, a single photon promotes the fluorophore from its ground state to an excited electronic state; the emission of a lower‐energy photon follows when the fluorophore relaxes. In TPE, two photons of approximately half the one‐photon energy are absorbed within an extremely short coincidence window. Under ordinary illumination conditions, the probability of two photons arriving within this window is negligible. Focused femtosecond pulsed lasers increase local photon densities sufficiently to make simultaneous two‐photon absorption experimentally practical [22]. The quadratic dependence of this process on the photon density means that the excitation is effectively limited to the focal point. This produces useful consequences where photobleaching and photodamage to the samples during the imaging process are restricted to the focal volume where the fluorophores are excited, thus leaving out‐of‐focus structures unperturbed [23]. Optical sectioning also arises naturally from nonlinear excitation without the need for a confocal pinhole. Furthermore, compared with the visible wavelengths used in single‐photon methods, NIR photons penetrate tissue more deeply.

### 2.2 Instrumentation for TPF Microscopy

A TPM can, in principle, be assembled around any laser‐scanning platform. Three elements are essential: an ultrafast pulsed laser, a high‐numerical‐aperture (NA) objective, and a sensitive photodetector. The main components and their roles are described below.

- *Pulsed laser source:* Mode‐locked Ti:sapphire lasers are standard, delivering femtosecond pulses at ~80 MHz repetition rates that are tunable over approximately 700–1100 nm. This tunability accommodates many fluorophores. Fiber lasers (typically Yb‐ or Er‐doped) offer a more compact, lower‐cost, and maintenance‐free alternative, generally at a fixed or narrowly tunable wavelength; because their pulses are formed through nonlinear amplification and spectral compression rather than free‐running mode‐locking in a bulk crystal, some fiber designs exhibit higher relative intensity noise and pulse‐to‐pulse timing jitter than Ti:sapphire oscillators, which can translate into greater shot‐to‐shot signal variability in photon‐counting applications such as FLIM [24, 25]. This noise penalty is design‐dependent rather than universal, and it has narrowed considerably in recent low‐noise fiber platforms, so noise specifications should be compared for the specific systems under consideration rather than assumed.
- *Beam scanning system:* Galvanometric mirrors raster‐scan the focused beam across the sample; beam expanders and steering optics shape and route the beam to the objective, and precise alignment is needed to maintain diffraction‐limited focusing across the field [25, 26].
- *Microscope objective:* High‐NA objectives concentrate excitation into a small focal volume and collect emitted photons efficiently, though they can introduce aberrations in scattering tissue and alter beam polarization, which is relevant for SHG measurements [27, 28].
- *Sample stage:* Motorized stages enable systematic scanning of large tissue areas/volumes and reproducible multilocation time‐lapse imaging.
- *Photodetectors:* Photomultiplier tubes (PMTs) and avalanche photodiodes (APDs) are standard; hybrid detectors (HyDs) combine low dark counts with high single‐photon detection efficiency for low‐signal work, while CCD cameras suit widefield rather than point‐scanning detection.
- *Dichroic mirrors and filters:* A dichroic mirror separates excitation light from emitted fluorescence, and bandpass filters select the spectral range for each detector channel while suppressing scattered excitation background.
- *Control and data acquisition:* Acquisition software coordinates laser scanning, stage movement, and detector readout, with real‐time processing enabling protocols such as automated mosaic imaging or triggered time‐lapse acquisition.

Beyond this component inventory, several parameters govern the practical performance of two‐photon imaging and merit particular attention. Because TPE efficiency scales with peak, rather than average, power, pulse duration and dispersion at the sample are critical: group‐delay dispersion introduced by the objective and other optics temporally broadens the pulse, lowering peak power and TPE probability, so dispersion pre‐compensation is often required for short (< 100 fs) pulses. Repetition rate sets the photon budget per pixel dwell time and, together with fluorophore excited‐state lifetime, determines the point at which increasing rate no longer improves signal (excited‐state saturation) while raising average‐power‐driven heating. Average excitation power governs linear photodamage and thermal loading, whereas peak power governs nonlinear photodamage and photobleaching; both must be minimized while retaining adequate signal. Objective NA and transmission jointly set the focal‐volume size, collection efficiency, and achievable resolution, with NIR transmission losses in some high‐NA optics reducing delivered peak power at the sample. Detection geometry also matters: non‐descanned, epi‐collected detection close to the objective maximizes collection of scattered emission photons in thick, turbid tissue, whereas descanned or trans‐detection geometries sacrifice scattered‐photon collection but can improve confocality. These parameters interact directly in the central trade‐off of two‐photon imaging: greater imaging depth requires higher incident power to compensate for scattering‐induced signal loss, but higher average and peak power at depth increase the risk of photodamage and photobleaching, so the achievable penetration depth in practice is set by the point at which further power increases no longer yield a net gain in usable signal relative to tissue damage.

### 2.3 Fluorescence Lifetime Imaging (FLIM)

Fluorescence intensity imaging has long been used to visualize molecular distributions in biological specimens; however, intensity‐based measurements are inherently influenced by fluorophore concentration, excitation power, optical attenuation, and detector sensitivity. These limitations motivated the development of FLIM, which maps the average time a fluorophore remains in its excited state before it returns to the ground state through photon emission—the fluorescence lifetime. This lifetime is sensitive to the molecular microenvironment of the fluorophore, which changes with factors such as the pH, ion concentration, oxygen tension, and proximity to energy acceptors in Förster resonance energy transfer (FRET) experiments. Unlike fluorescence intensity, fluorescence lifetime is comparatively insensitive to fluorophore concentration and excitation‐power fluctuations, which makes it a useful quantitative parameter for probing the biochemical and biophysical states of tissues; however, this insensitivity is not absolute. Concentration‐dependent interactions such as self‐quenching and homo‐FRET, inner‐filter reabsorption at high optical densities, limited photon statistics, detector timing response, and the choice of decay‐fitting model can each introduce systematic shifts in the measured lifetime, so apparent independence from concentration and excitation power holds only within the regime where these effects are negligible [29]. This consideration is particularly relevant to two‐photon FLIM, where the low two‐photon absorption cross‐sections of endogenous fluorophores such as NAD(P)H yield comparatively few photons per pixel within practical dwell times, making the measured lifetime more susceptible to photon‐statistical noise and to the specific fitting algorithm used than to fluorophore concentration itself. Since its introduction in the late 20th century, FLIM has evolved from a niche spectroscopic technique into an indispensable tool for biomedical imaging. Early applications focused primarily on exogenous fluorescent probes, whereas contemporary FLIM systems increasingly exploit endogenous fluorophores, such as NAD(P)H and FAD, to characterize cellular metabolism, redox state, protein binding, and tissue heterogeneity in a label‐free manner. This transition has established FLIM as a powerful complement to nonlinear optical modalities, particularly in applications requiring functional information beyond structural contrast. Consequently, FLIM is now routinely integrated with confocal, multiphoton, and polarization‐sensitive imaging platforms to provide simultaneous morphological and biochemical characterization of biological systems [30, 31, 32]. Two principal approaches have emerged for fluorescence lifetime measurement: time‐domain and frequency‐domain FLIM. Time‐domain FLIM, particularly time‐correlated single‐photon counting (TCSPC), remains the most widely adopted implementation owing to its high temporal resolution and sensitivity. In this approach, ultrashort laser pulses excite the sample, and the arrival time of each emitted photon is recorded relative to the excitation pulse. The accumulation of millions of photon arrival events reconstructs the fluorescence decay profile, from which one or more lifetime components are extracted through mathematical fitting [33]. In contrast, frequency‐domain FLIM employs sinusoidally modulated excitation and estimates fluorescence lifetimes from the phase delay and demodulation of the emitted signal. Although frequency‐domain systems can offer rapid full‐field acquisition and are attractive for high‐throughput imaging applications, they often provide lower temporal precision than TCSPC‐based implementations do, particularly when complex multicomponent decays are resolved (Figure 2) [35].

**FIGURE 2:**
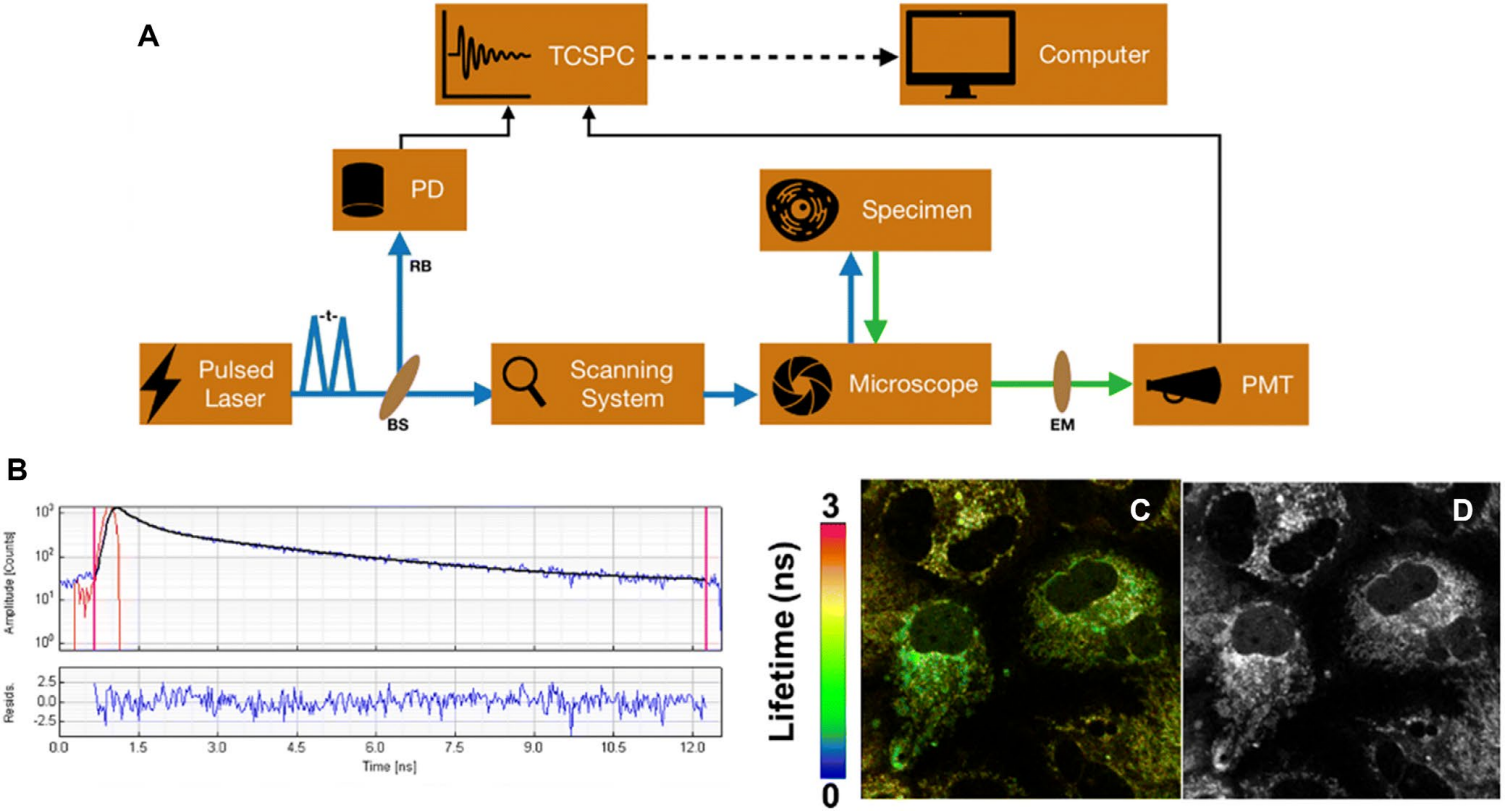
(a) Typical 2P‐FLIM setup using time‐correlated single‐photon counting (TCSPC). BS, beam splitter; EM, emission filter; PD, photodiode; PMT, photomultiplier tube; RB, reference beam. (b) Biexponential NAD(P)H fluorescence decay with *χ* ^2^≈1. (c) Color‐coded NAD(P)H lifetime map in Huh7 cells. (d) Fluorescence intensity image of NAD(P)H. Scale bar = 10 μm. Adapted with permission from [34].

Despite its advantages, FLIM is not without limitations. Acquisition speed is fundamentally constrained by photon statistics, often necessitating prolonged imaging times for accurate lifetime estimation in weakly fluorescent samples. Moreover, fluorescence decay analysis can be computationally demanding, particularly when multiexponential fitting models are employed. Additional challenges include photobleaching, phototoxicity, and limited imaging depth in conventional single‐photon systems, which become increasingly problematic in thick and highly scattered biological specimens. These constraints motivated the integration of FLIM with multiphoton excitation, resulting in two‐photon FLIM (2P‐FLIM), which combines functional lifetime contrast with the intrinsic optical sectioning capabilities of nonlinear microscopy [36]. By employing NIR femtosecond excitation, 2P‐FLIM confines fluorescence generation to the focal volume, thereby reducing out‐of‐focus photobleaching and photodamage while enabling imaging depths of several hundred micrometers in biological tissue specimens. The use of longer excitation wavelengths also mitigates scattering and further improves penetration depth compared with single‐photon excitation. These advantages have made 2P‐FLIM particularly valuable for investigating dynamic biological processes in intact tissues, including tumor metabolism, neuronal signaling, stem cell differentiation, and tissue regeneration. Nevertheless, trade‐offs remain: the imaging depth is still constrained by optical scattering and aberrations, the acquisition rates are limited by the photon collection efficiency, and the requirements for ultrafast laser sources and sensitive timing electronics increase the system complexity and cost [29, 37, 38]. In the context of nonlinear optical microscopy, FLIM is unique because it provides functional and metabolic information that complements the structural insights obtained from techniques such as SHG and TPF. The integration of these modalities has facilitated the development of multimodal imaging platforms capable of simultaneously interrogating tissue architecture, extracellular matrix (ECM) organization, and cellular metabolism, thereby offering a more comprehensive understanding of physiological and pathological processes [39, 40].

#### 2.3.1 Photon Timing Principles

Photon timing in FLIM depends on measuring the delay between an excitation laser pulse and the arrival of an emitted photon at the detector. A timing circuit receives trigger pulses from the laser and stops pulses from the detector, computing the time difference for each detected photon. These time differences are sorted into discrete ‘time bins’, with the bin width and total number chosen to span approximately five fluorescence lifetimes so that the entire decay can be captured before the signal merges with the background. Repeating this measurement across thousands of photons builds a decay histogram that is then analyzed to extract the lifetime [36]. The accuracy of the measurement is limited by the instrument response function (IRF), which describes how the detection system itself distorts an instantaneous signal. The total IRF width combines contributions from the optical pulse duration, detector transit time spread, and electronic timing jitter:

$$\Delta {t^{2}}_{\mathrm{IRF}}=\Delta {t^{2}}_{\text{optical}}+\Delta {t^{2}}_{\mathrm{tts}}+\Delta {t^{2}}_{\text{jitter}}$$

Here, *Δt*_optical_ is the excitation pulse width, *Δt*_tts_ is the detector transit time spread, and *Δt*_jitter_ is the electronic timing jitter. In two‐photon systems, pulse widths in the femtosecond range make *Δt*_optical_ negligible; timing electronics can limit the jitter to approximately 1 ps in optimized systems.

Autofluorescence from endogenous fluorophores is often analyzed with a biexponential decay model:

$$I\left(t\right)=\alpha_{1}e^{\left(-t/\tau_{1}\right)}+\alpha_{2}e^{\left(-t/\tau_{2}\right)}$$

where (*τ*_1_, *α*_1_) and (*τ*_2_, *α*_2_) are the lifetimes and amplitudes of the fast and slow components, respectively. The amplitude‐weighted mean lifetime *τ*_*m*_ = (*a*_1_ *τ*_1_ + *a*_2_ *τ*_2_)/(*a*_1_ + *a*_2_) provides a single summary parameter often used for metabolic mapping. The phasor approach offers an alternative, model‐free analysis: the decay curve is Fourier transformed to yield coordinates *G* and *S* in the phasor space, where single‐lifetime components fall on the semicircle and mixtures lie within it [29, 37, 38, 39, 40]. Figure 3 shows the schematic of time domain and frequency domain lifetime measurement.

**FIGURE 3:**
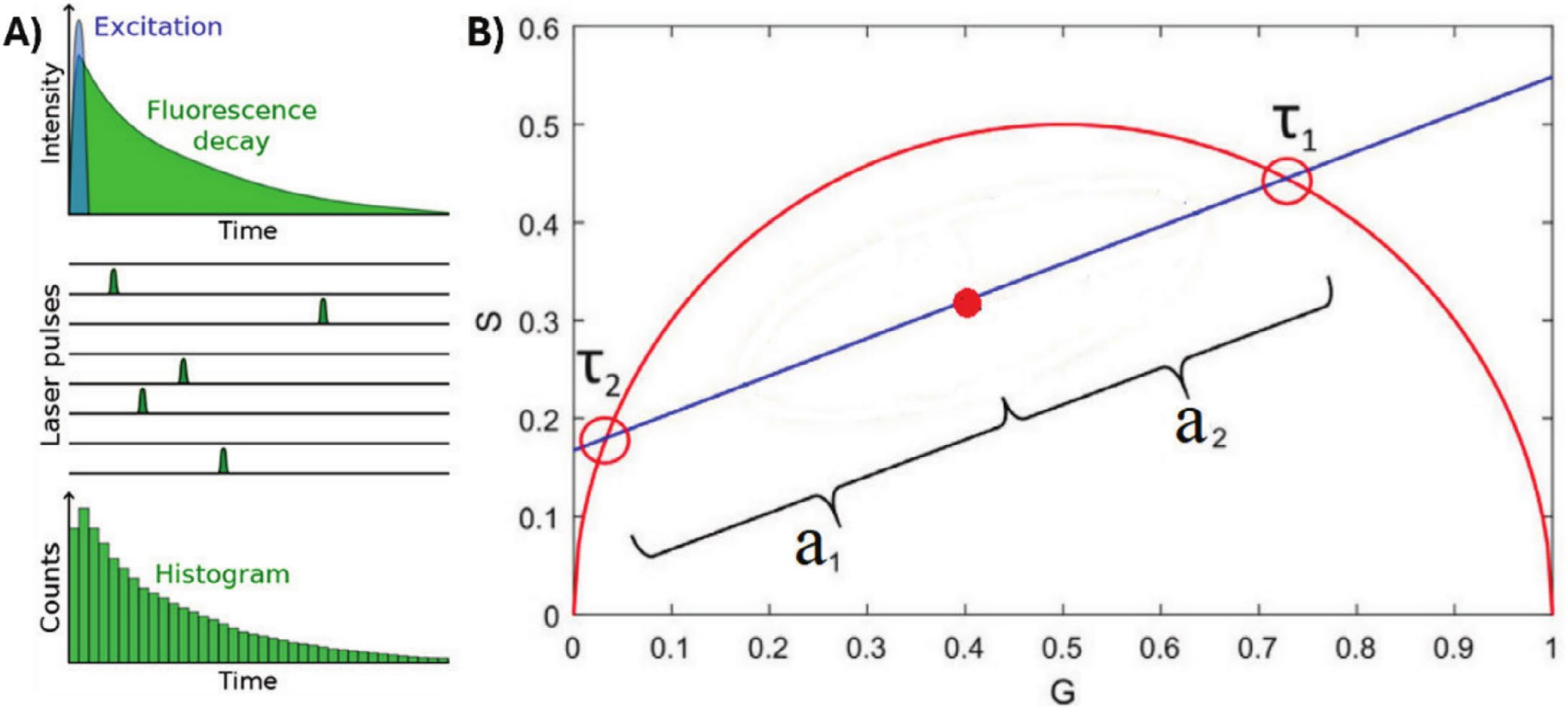
(A) Schematic of TCSPC photon arrival timing: Repeated excitation pulses and recorded photon arrival times are used to construct a fluorescence decay histogram. Reproduced with permission from [36]. (B) Phasor plot showing single‐fluorophore lifetimes (*τ*_1_, *a*_1_) and a two‐component mixture (*τ*_2_, *a*_2_). Adapted from [41].

### 2.4 Applications of TPF Microscopy

The application landscape of TPM has evolved in parallel with advances in instrumentation where, with each successive technological innovation, the major limitations of early systems, namely, imaging depth, optical aberrations, acquisition speed, and restricted functional capabilities, were also addressed. The initial TPM studies focused primarily on structural imaging of fixed and superficial specimens. However, improvements in laser technology, detector sensitivity, and optical engineering have transformed TPM into a versatile platform for long‐term, high‐resolution investigations of living tissues.

One of the earliest challenges in in vivo TPM was maintaining image quality at increasing depths. Biological tissues introduce significant scattering and wavefront distortions that degrade spatial resolution and reduce signal collection efficiency. Adaptive optics (AO), originally developed for astronomical imaging, has emerged as an effective solution for compensating for tissue‐induced aberrations. For example, Qin et al. demonstrated an AO‐assisted TPM system capable of restoring near‐diffraction‐limited imaging in the mouse retina, enabling visualization of retinal microstructures and axonal degeneration with significantly improved contrast and resolution [42]. In another study, Yamaguchi et al. integrated AO in TPM using a spatial light modulator to compensate for optical aberrations introduced by refractive index mismatches at specimen surfaces. By calculating specimen‐induced wavefront distortions through three‐dimensional ray tracing and applying the corresponding phase corrections, this approach restored the excitation point spread function, thereby improving the image quality. More importantly, the AO‐integrated TPM demonstrated enhanced fluorescence intensity and image contrast in biological samples, including multicellular tumor spheroids and deep cortical regions of living mouse brains with curved surfaces [43]. These improvements enable more reliable deep‐tissue imaging and facilitate the visualization of fine cellular structures, highlighting the potential of AO‐enhanced TPM for studying dynamic physiological processes in complex biological specimens.

The demand for chronic and longitudinal imaging has further driven TPM development. Conventional histological approaches require tissue excision and provide only static snapshots of dynamic biological processes. TPM overcomes this limitation by enabling repeated imaging of the same sample over extended periods. Yang et al. leveraged this capability to monitor neuroinflammatory responses surrounding intracortical implants in awake mice over several weeks, revealing persistent alterations in microvascular architecture and immune cell recruitment that would have been inaccessible using endpoint histology [44]. Similarly, label‐free multimodal imaging approaches combining two‐photon autofluorescence and SHG have enabled the simultaneous assessment of cellular metabolism and ECM remodeling. Zhuo et al. demonstrated the utility of this strategy in monitoring wound healing, where metabolic changes and collagen reorganization could be tracked concurrently in vivo (Figure 4) [45]. One of the other major challenges of conventional TPM is its limited temporal resolution. The sequential point‐scanning approach, while providing high spatial resolution, results in relatively slow image acquisition, making monitoring rapid biological processes difficult. To address this, recent efforts have focused on optimizing fluorophores, scanning architectures, and detection schemes. Takezaki et al. developed a fluorescent nanoprobe specifically tailored for deep‐tissue TPM, facilitating rapid imaging of cerebrovascular networks while maintaining sufficient signal intensity at depth [46, 47]. Such advances are particularly relevant for functional neuroimaging, where capturing transient physiological processes necessitates a careful balance between acquisition speed, signal‐to‐noise ratio, and phototoxicity.

**FIGURE 4:**
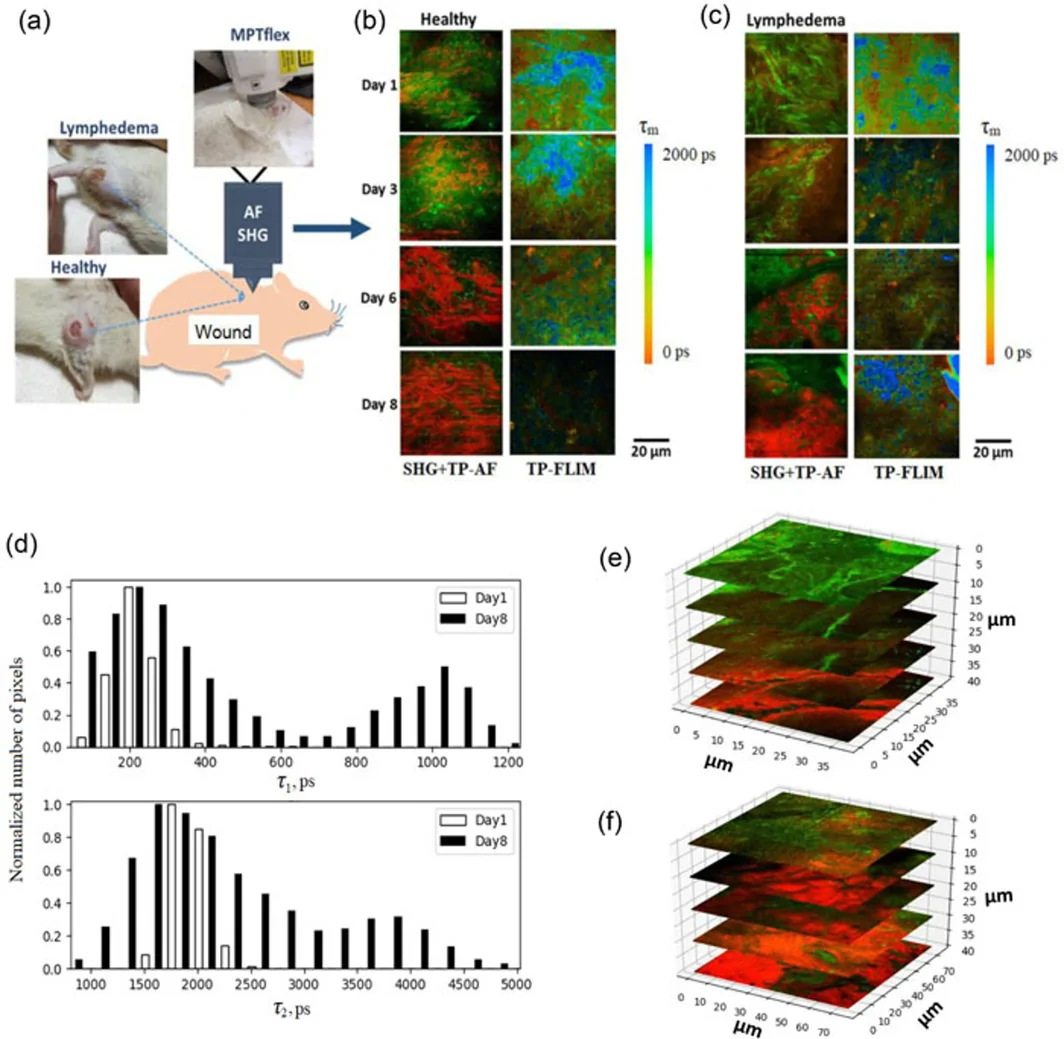
In vivo wound healing imaging by multiphoton microscopy. (a) Imaging scheme. (b and c) SHG (red) + TP‐AF (green) and TP‐FLIM images from healthy and lymphedematous tissue, respectively. (d) Lifetime distributions on Days 1 and 8. (e and f) 3D SHG + TP‐AF reconstructions on Day 8. Adapted from [45].

Beyond imaging, TPM has increasingly become an intervention platform. The confinement of TPE to the focal volume allows highly localized photochemical reactions, enabling precise manipulation of cells and tissues. Reviews by Luu et al. have highlighted the emergence of two‐photon photostimulation and uncaging techniques, which permit simultaneous observation and perturbation of biological systems [48]. Building upon these principles, Yamaguchi et al. demonstrated TPM‐guided laser ablation within cortical layer V by optimizing the beam diameter and immersion media, illustrating the potential for targeted microsurgery deep within intact tissue [49] (Figure 5). These developments underscore a broader transition in the field—from passive observation to active interrogation of biological function. The convergence of TPM with optogenetics represents another significant milestone in its technological progression. By coupling TPE with genetically encoded actuators, investigators have achieved cellular and even subcellular specificity in neural stimulation. Tong et al. reported single‐cell optogenetic activation in vivo using TPE, whereas Shymkiv and Yuste developed holographic TPM platforms capable of simultaneous imaging and patterned stimulation across neuronal populations [50, 51, 52]. Such systems provide unprecedented opportunities to establish a relationship between neural activity and animal behavior, thus positioning TPM as a central tool in systems neuroscience. More recently, efforts have focused on improving the accessibility and translational potential of TPM. Traditional benchtop systems remain expensive, bulky, and difficult to deploy in clinical environments. Fiber‐based and miniaturized TPM architectures have therefore attracted considerable attention. Zhu et al. reviewed advances in fiber‐optic TPM systems, highlighting their potential for minimally invasive endoscopic imaging and intraoperative applications [53]. Concurrently, specialized instrument designs continue to expand TPM into challenging experimental settings. Collectively, these developments illustrate technological progress in TPM, from a modality primarily used for high‐resolution structural imaging to a multifunctional platform capable of deep‐tissue visualization, longitudinal monitoring, high‐speed acquisition, precise tissue manipulation, and closed‐loop functional interrogation. Future advances are expected to further integrate adaptive optics, computational imaging, artificial intelligence, and multimodal nonlinear microscopy, ultimately enhancing the translational utility of TPM in both basic research and, more importantly, clinical settings.

**FIGURE 5:**
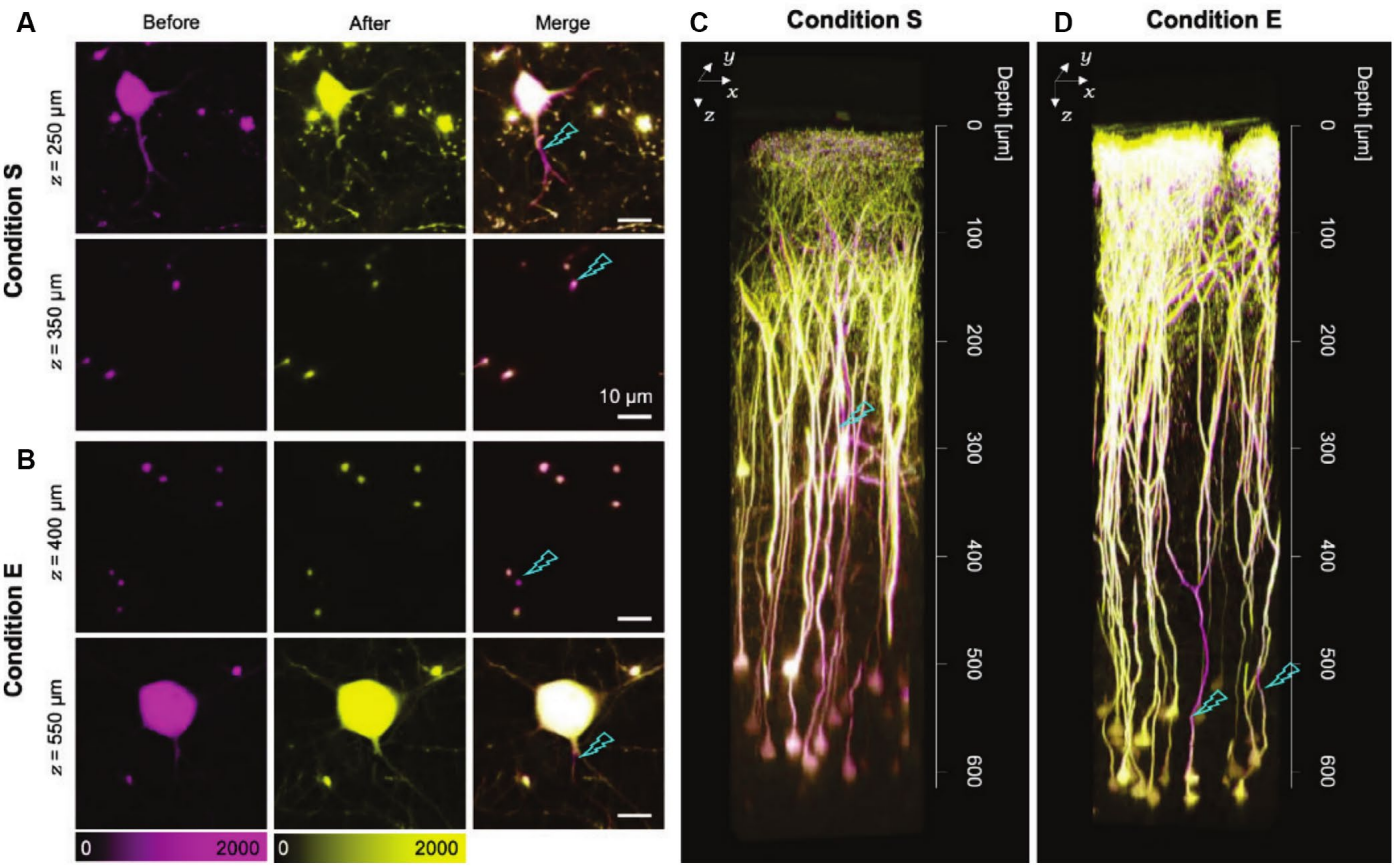
Two‐photon focal laser ablation in cortical layer V. Before/after/merged fluorescence images (conditions S and E) and 3D reconstructions showing ablation depth and precision. (a, b) Fluorescence images obtained before and after the laser ablation under (a) condition S and (b) condition E. The right panels show the merged images. (c, d) 3D reconstructed images merged before focal laser irradiation and after 1 day under (c) condition S and (d) condition E. Reproduced from [49] with permission from Wiley.

## 3 SHG Microscopy

SHG microscopy occupies a unique position within the family of nonlinear optical imaging techniques. Unlike fluorescence‐based modalities, which rely on photon absorption and subsequent emission, SHG is an elastic scattering process in which two photons of identical frequency interact coherently with a noncentrosymmetric medium to generate a single photon at exactly twice the incident frequency. Since no real electronic transition is involved, compared with conventional fluorescence imaging, SHG does not deposit energy into the sample, rendering the technique intrinsically resistant to photobleaching and significantly less phototoxic [54].

The emergence of SHG microscopy was largely driven by limitations inherent to fluorescence imaging. While fluorescence microscopy provides excellent molecular specificity, it typically requires exogenous labels, suffers from photobleaching, and often lacks sensitivity to the ultrastructural organization of tissues. SHG addresses these shortcomings by exploiting endogenous contrast arising from ordered molecular assemblies. Consequently, SHG is particularly sensitive to fibrillar collagen, myosin, microtubules, and other noncentrosymmetric structures, enabling label‐free visualization of tissue architecture with submicron resolution [55, 56]. The history of SHG imaging spans several decades. Experimental SHG was first demonstrated in 1961 following the invention of the ruby laser, and its electromagnetic theory was formalized by Bloembergen and Pershan in 1962 [54, 57]. However, the application of SHG to biological imaging did not emerge until several decades later. In 1986, Freund et al. demonstrated SHG from collagen in the rat tail tendon, establishing the potential of this technique for probing biological organization [58]. Despite this early success, widespread adoption was limited by the availability of suitable ultrafast laser sources and detection technologies. The development of femtosecond Ti:sapphire lasers in the 1990s, coupled with advances in laser‐scanning microscopy, marked a turning point for SHG imaging. These technological improvements enabled efficient signal generation with minimal photodamage and facilitated the integration of SHG into multiphoton microscopy platforms. Since then, SHG has evolved from a niche optical phenomenon into an indispensable tool for investigating ECM remodeling, fibrosis, cancer progression, wound healing, and musculoskeletal disorders [56]. Importantly, the evolution of SHG microscopy has mirrored the increasing demand for quantitative tissue characterization. Early implementations provided qualitative structural information, whereas modern SHG systems incorporate polarization control, adaptive optics, and computational image analysis to extract quantitative metrics related to collagen and organization. These developments have positioned SHGs not only as visualization tools but also as functional biomarkers of tissue health and disease [59, 60, 61]. The subsequent sections discuss the photophysics and other physical mechanisms of SHG signals along with the application of SHG microscopy in the biomedical field.

### 3.1 Photophysics of SHG

The SHG process can be understood by analogy with a nonlinear oscillator. An ideal harmonic oscillator driven at frequency *ω* responds only at *ω*. An anharmonic oscillator, however, responds at the drive frequency and at its harmonics (2*ω*, 3*ω*, etc.). In nonlinear optical media, the electronic polarization of molecules driven by an intense optical field behaves analogously. For a strong external field *E*, the polarization *P* can be written as a power series:

$$P=\varepsilon_{0}\left[\chi^{-1}E+\chi^{-2}E^{2}+\chi^{-3}E^{3}+\cdots \right]$$

Here, *χ* ^−1^ is the linear susceptibility, and *χ* ^−2^ and *χ* ^−3^ are the second‐ and third‐order nonlinear susceptibilities, respectively. SHG arises from *χ* ^−2^, which is nonzero only in media lacking inversion symmetry (noncentrosymmetric materials). In a centrosymmetric medium, equal and opposite polarization contributions cancel, and no SHG is produced. The second‐order nonlinear polarization at a frequency of 2*ω* is given in the frequency domain by the tensor relation:

$$\begin{array}{c} P^{-2}\left(\mathrm{\omega n}+\mathrm{\omega m}\right)=\varepsilon_{0}\sum_{\mathrm{jk}}\sum \left(\mathrm{nm}\right){\chi^{-2}}_{\mathrm{ijk}}\left(\mathrm{\omega n}+\mathrm{\omega m},\mathrm{\omega n},\mathrm{\omega m}\right)E_{J}\left(\mathrm{\omega n}\right)E_{k}\left(\mathrm{\omega m}\right) \end{array}$$

where *i*, *j*, and *k* are Cartesian indices and the sum retains only contributions at the output frequency. Only specific symmetry‐allowed tensor elements contribute to a given material, so the pattern of *χ* ^−2^ elements serves as a fingerprint of molecular symmetry.

#### 3.1.1 Contrast Mechanism

The contrast observed in SHG microscopy arises from the coherent addition of second‐harmonic fields generated by ordered molecular assemblies. SHG contrast reflects the degree of molecular order in the sample. Disordered distributions of noncentrosymmetric molecules, such as those in isotropic solutions, produce incoherent second‐order scattering (hyper Rayleigh scattering, HRS) rather than coherent SHG. Coherent SHG builds up when many molecular dipoles are arranged in a locally ordered, noncentrosymmetric fashion: contributions from individual second‐harmonic response emitters (SHREs) add constructively. The bulk second‐order susceptibility *χ* ^−2^ is related to the molecular hyperpolarizability *β* and the number of coherently contributing emitters *N*_s_ as follows:

$$\chi^{-2}=N_{s}\left\langle \beta \right\rangle$$

Here, ⟨*β*⟩ is the orientational average of *β*. Biological SHG sources are dominated by type I and II fibrillar collagens and by myosin within actomyosin arrays; elastin and other structurally disordered proteins do not generate SHG because they lack the required long‐range molecular ordering [62].

#### 3.1.2 Phase Matching and Directionality

One of the defining characteristics of SHG is its dependence on phase matching. In ideal nonlinear crystals, efficient SHG requires the phase velocity of the fundamental wave to match that of the second harmonic. The wave‐vector mismatch *Δk* = 2 *k*_1_ − *k*_2_ determines the coherence length *L*_*c*_ = 2*π*/*Δk* over which fundamental and harmonic waves remain in phase. When *Δk* = 0, perfect phase matching gives an infinite coherence length, and the SHG signal propagates entirely forward. In biological tissues, perfect phase matching is generally not achievable because the molecular arrangement and birefringence do not satisfy the conditions required for type I matching. A nonzero *Δk* reduces the conversion efficiency and shifts some SHG signals in the backward direction according to the following equation:

$$I_{\mathrm{SHG}}\propto \sin^{2}\left(\text{nΔkL}/2\right)$$

where *n* is an integer and *L* is the coherence length. Smaller *Δk* values produce stronger forward SHG; larger *Δk* values favor backward emission. In practice, measuring the forward‐to‐backward SHG ratio provides information about collagen fibril diameter and packing. The Gouy phase shift—a *π* phase change that occurs as a Gaussian beam passes through focus—also affects phase matching at the microscopic level and reduces the SHG efficiency for very small focal volumes.

#### 3.1.3 SHG Signal Generation in Biological Tissue

In biological samples, the strict phase‐matching constraints that govern crystal‐based SHG are relaxed. Tissues tolerate a degree of phase mismatch, and the resulting SHG signal, although weaker than in a perfectly phase‐matched geometry, is sufficient for imaging. The dominant SHG sources in tissue are type I and type II fibrillar collagen, whose hierarchical triple‐helix structure provides the ordered noncentrosymmetric packing needed for coherent second‐harmonic emission and myosin filaments in striated muscle. Disordered proteins such as elastin do not contribute, so the SHG images show collagen and myosin with inherent chemical selectivity. The signal strength depends on the laser pulse energy, repetition rate, and objective NA and can be optimized by adjusting these parameters. Importantly, SHG should not be viewed as a standalone modality. Its greatest utility often arises when it is combined with complementary nonlinear techniques. For instance, simultaneous SHG and two‐photon autofluorescence imaging allows investigators to correlate ECM organization with cellular metabolism, providing a more comprehensive understanding of tissue physiology and pathology. This multimodal paradigm has become increasingly prevalent in studies of cancer, fibrosis, and tissue regeneration [63].

### 3.2 SHG Microscopic Instrumentation

SHG microscopy typically uses the same femtosecond Ti:Sa laser and scanning optics as TPM, operating at an 800 nm fundamental wavelength to generate SHG at 400 nm (Figure 6). Narrowband SHG filters remove the fundamental before detection by PMTs operating in single‐photon counting mode. Both forward (transmission) and backward (epi) geometries are commonly used; backward detection is more practical for thick tissues and in vivo measurements. For polarization‐resolved SHG, half‐wave and quarter‐wave plates rotate the excitation polarization to multiple angles, and a linear analyzer is placed before the detector selects the polarization of the SHG signal. This arrangement allows the second‐order susceptibility tensor *χ* ^−2^ to be measured as a function of the excitation and detection polarization angle, yielding quantitative information about molecular orientation. Several calibration steps are needed to obtain accurate polarization‐resolved SHG data. First, the optical elements introduce ellipticity into what should be linearly polarized excitation; a quarter‐wave plate is used to precompensate this ellipticity at each rotation angle, verified with a polarizing beam splitter and power meter. Second, the polarization‐dependent reflectivity of the dichroic mirror causes the excitation power to vary with respect to the angle; this is corrected by adjusting the laser power at each angle. Third, high‐NA objectives can scramble the polarization state; moderate‐NA objectives (e.g., 0.8 NA) are often preferred for polarization measurements to limit this effect. A portable polarization module can be added to an existing commercial microscope without permanent modification, preserving the versatility of the instrument for other applications [64].

**FIGURE 6:**
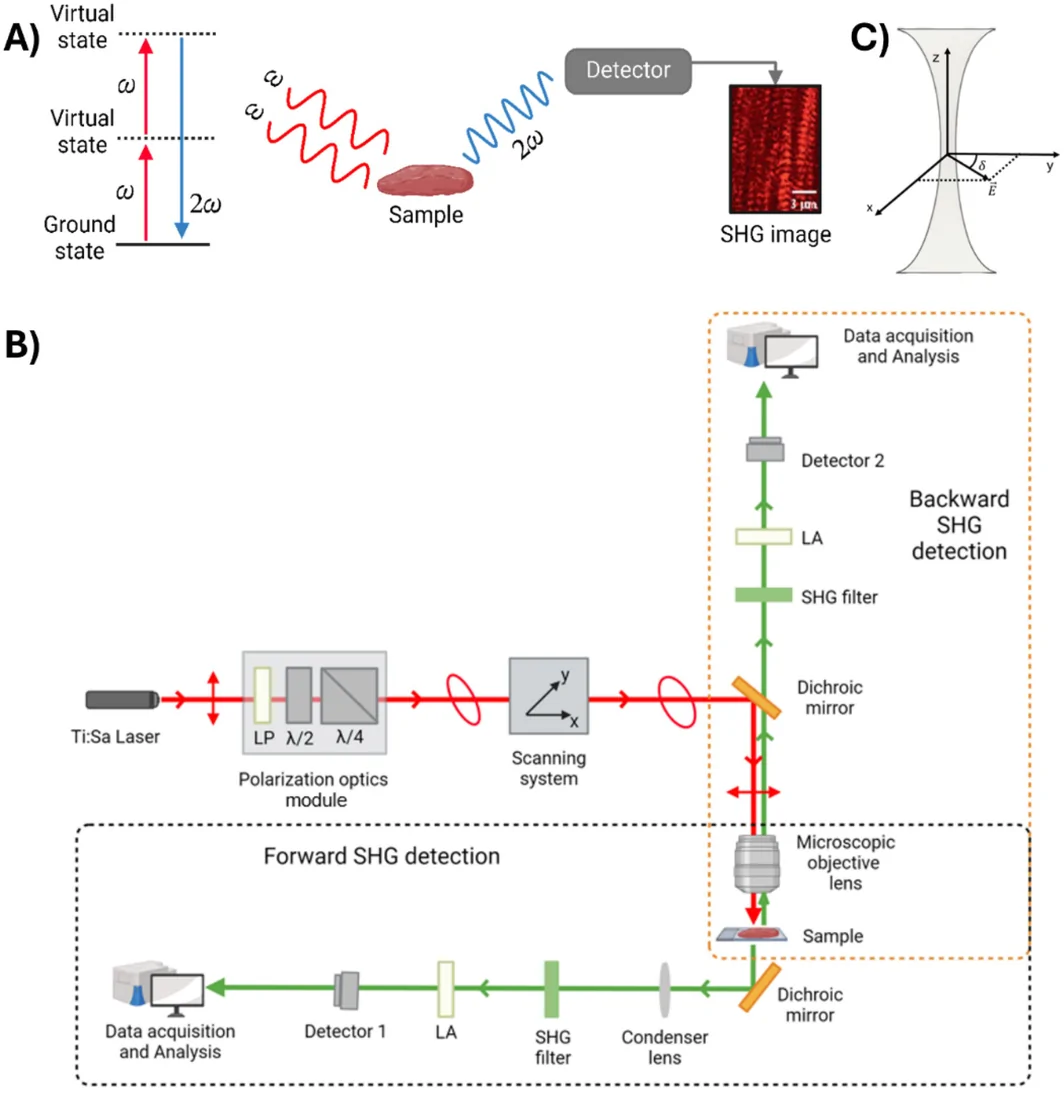
(A) Energy diagram for SHG in a noncentrosymmetric sample. (B) Simplified schematic of an SHG/polarization‐resolved SHG setup. LA, linear analyzer; LP, linear polarizer; *λ*/2, half‐wave plate; *λ*/4, quarter‐wave plate; Ti:Sa, titanium sapphire laser. (C) Coordinate system for calculating the SHG intensity from a cylindrically symmetric SHRE distribution.

### 3.3 Polarization‐Resolved SHG Microscopy

Although conventional SHG microscopy provides excellent contrast for noncentrosymmetric structures, its principal limitation lies in the fact that image intensity alone offers limited insight into molecular organization. Two collagen‐rich tissues can produce comparable SHG intensities despite exhibiting markedly different fibrillar orientations and ultrastructural arrangements. This limitation motivated the development of polarization‐resolved SHG (P‐SHG) microscopy, which exploits the polarization dependence of the second‐order susceptibility tensor $\chi^{2}$ to extract quantitative information regarding molecular orientation and symmetry. The foundations of P‐SHG were established in the early 2000s through the work of Stoller, Campagnola, and colleagues, who demonstrated that polarization‐dependent measurements could reveal collagen architecture beyond the capabilities of conventional SHG imaging [65, 66, 67]. Since then, P‐SHG has emerged as one of the most widely adopted quantitative extensions of SHG microscopy, particularly for studies involving collagen remodeling, fibrosis, and cancer.

P‐SHG microscopy measures the dependence of the SHG intensity on the excitation and detection polarization angles to map the $\chi^{2}$ tensor of a sample. For cylindrically symmetric collagen fibrils, the ratio of two independent tensor components, often written as *χ* ^1^ = *χ* ^2^_*zzz*_/*χ* ^2^_*zxx*_, indicates the average tilt angle of the peptide group dipoles relative to the fibril axis. This parameter differs between collagen types I and III, allowing P‐SHG to distinguish fibril populations that appear identical in standard SHG images. For instance, P‐SHG has been used to quantify the relative content of collagen types I and III in ovarian tissue, where the balance of these fibril types shifts with the reproductive cycle [64] (Figure 7). The ratios of *χ* ^−2^ tensor elements could function as biomarkers for pathological matrix remodeling in fibrosis, wound healing, and tumor progression.

**FIGURE 7:**
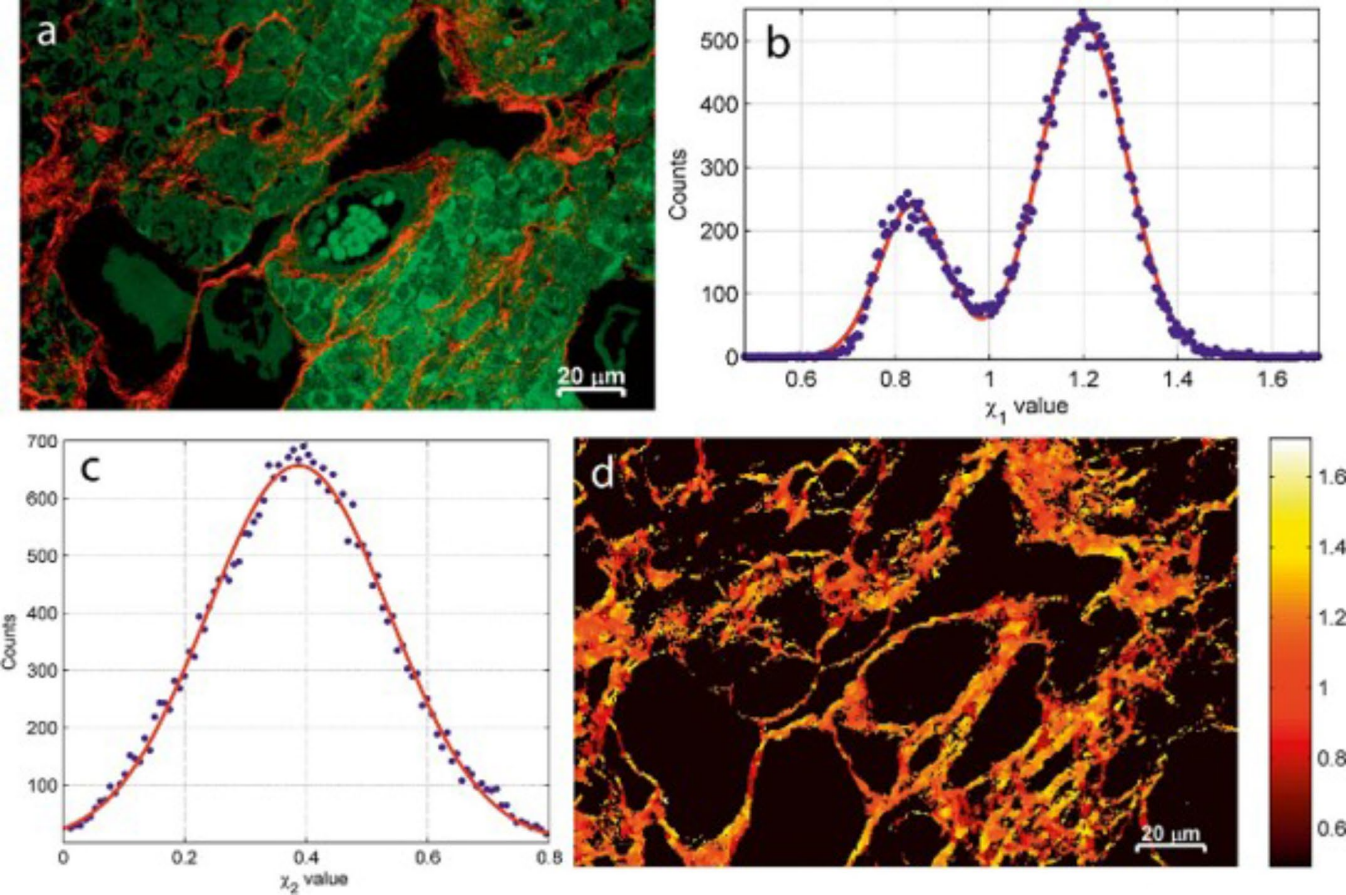
P‐SHG imaging of ovarian tissue. (a) SHG image; (b) histogram of *χ*_1_ values; (c) *χ*_2_ histogram; (d) *χ*_1_ map showing the spatial distribution of collagen types I and III. Adapted from [64].

However, P‐SHG is accompanied by several practical limitations. Accurate measurements require rigorous polarization calibration, as optical components—including dichroic mirrors, wave plates, and high numerical aperture objectives—can introduce polarization distortions. Furthermore, the acquisition times are also longer than those of conventional SHG because multiple polarization states must be sequentially recorded. Nevertheless, the ability of P‐SHG to quantify molecular orientation has transformed SHG from a qualitative imaging technique to a quantitative tool for tissue characterization. Consequently, P‐SHG represents a significant milestone in the evolution of nonlinear microscopy, bridging the gap between structural imaging and quantitative assessment of tissue ultrastructure.

### 3.4 SHG Circular Dichroism (SHG‐CD)

While P‐SHG provides information regarding molecular orientation within the imaging plane, it remains relatively insensitive to molecular chirality and out‐of‐plane organization. To address these limitations, researchers introduced circular polarization‐based approaches, leading to the development of SHG‐CD. Unlike conventional circular dichroism spectroscopy, which relies on differential absorption of left‐ and right‐handed circularly polarized light, SHG‐CD arises from the nonlinear interaction of circularly polarized excitation with chiral, noncentrosymmetric structures [68, 69]. SHG‐CD extends polarization analysis to left‐ and right‐handed circular polarization (LCP and RCP). The SHG‐CD signal is defined as follows:

$$I_{\mathrm{CD}-\mathrm{SHG}}=\left[I{\left(2\omega \right)}_{\mathrm{LCP}}-I{\left(2\omega \right)}_{\mathrm{RCP}}\right]/\left[\left(I{\left(2\omega \right)}_{\mathrm{LCP}}+I{\left(2\omega \right)}_{\mathrm{RCP}}\right)/2\right]$$

The absolute value of *I*_CD‐SHG_ indicates whether the fibrils are oriented within the image plane (small values) or tilt out of the plane (large values), providing three‐dimensional orientation information that is inaccessible from the SHG intensity image alone [70]. The contrast enhancement relative to linear CD can reach three orders of magnitude, reflecting the greater sensitivity of second‐order susceptibility to molecular chirality at the macroscopic level [59, 71].

Seminal studies by Schmeltz et al. demonstrated the utility of SHG‐CD for imaging collagen organization in human corneal tissues, whereas Lee et al. extended its application to characterize collagen type I and other chiral biological structures [59, 70]. More recently, Zhuo et al. employed SHG‐CD to investigate starch granules, illustrating the broader applicability of the technique beyond connective tissue imaging [60].

Despite its advantages, SHG‐CD remains technically demanding. The measured signal is highly sensitive to residual ellipticity introduced by the optical train and can be influenced by birefringence and scattering within the sample [72]. Furthermore, interpretation of SHG‐CD measurements often requires computational modeling to distinguish genuine chiral effects from orientation‐dependent artifacts. Consequently, SHG‐CD has largely remained a specialized technique confined to laboratories with extensive expertise in nonlinear optics. Nevertheless, SHG‐CD represents an important advancement in the progression of SHG microscopy, extending the modality from two‐dimensional orientation mapping toward three‐dimensional characterization of molecular chirality.

### 3.5 Stokes Vector‐Resolved SH Microscopy

Although P‐SHG and SHG‐CD provide valuable information regarding molecular orientation and chirality, they characterize only selected aspects of the polarization response. Modern polarimetric imaging increasingly seeks to capture the complete polarization state of the emitted SHG signal, motivating the development of Stokes vector‐ and Mueller matrix‐resolved SHG microscopy. The Stokes vector formalism describes any polarization state, including partially polarized and depolarized light, through four parameters (*S*_0_, *S*_1_, *S*_2_, and *S*_3_) that represent total intensity, linear polarization along the horizontal/vertical direction, linear polarization at ±45°, and circular polarization, respectively. A four‐channel Stokes polarimeter splits the SHG signal into four polarization channels and reconstructs the full Stokes vector from the measured intensities. This is more general than single‐analyzer measurements because it characterizes the polarization state of the outgoing SHG field rather than only its projection onto one axis [61, 73, 74, 75]. From the measured Stokes vector, the degree of polarization (DOP), degree of linear polarization (DOLP), and degree of circular polarization (DOCP) can be computed as follows:

$$\mathrm{DOP}=\surd \left({S_{1}}^{2}+{S_{2}}^{2}+{S_{3}}^{3}\right)/S_{0}$$

$$\text{DOLP}=\surd \left({S_{1}}^{2}+{S_{2}}^{2}\right)/S_{0}$$

$$\text{DOCP}=S_{3}/S_{0}$$

The instrument matrix *A*_4 × 4_, which relates the Stokes output vector to the measured intensity vector, is calibrated with a polarization state generator (PSG) [67]. Mueller matrix decomposition, obtained by sequentially varying the input polarization states, characterizes the full polarimetric response of the sample. When applied to noncentrosymmetric biological structures such as collagen networks, skeletal muscle myosin, keloid tissue, starch granules, and birefringent crystals, Stokes‐resolved SHG provides quantitative maps of DOP, DOLP, DOCP, and polarization anisotropy [76, 77, 78, 79] (Figure 8).

**FIGURE 8:**
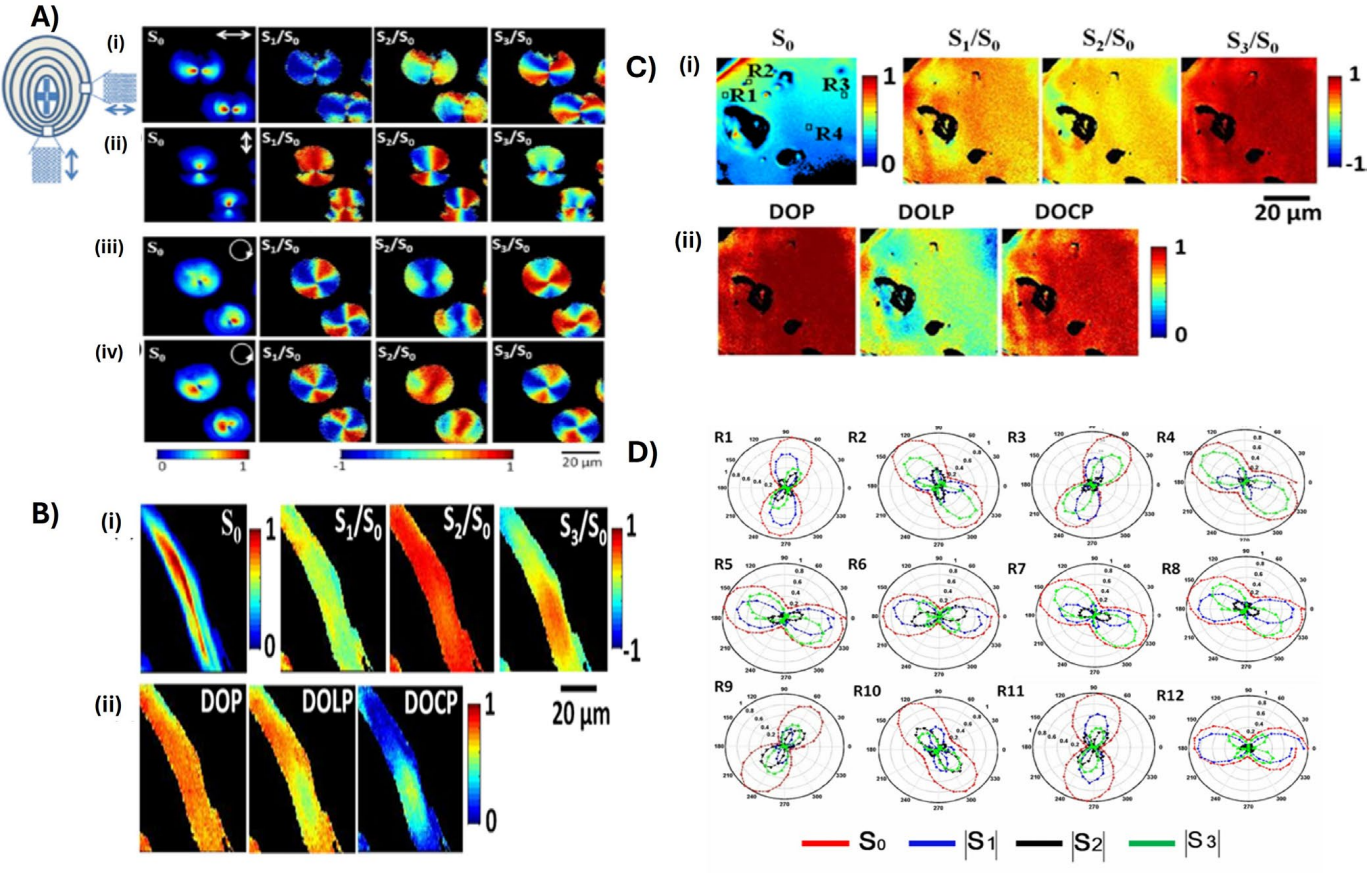
Stokes vector SHG analysis of starch granules showing the two‐lobe structure in the total SH intensity (*S*_0_) image, which reflects the radial alignment of the SHG‐active amylopectin crystals. A) SHG response from starch granules B) type‐I collagen C) KDP micro‐crystals. D) Graph shows the absolute values of Stokes parameters as a function of incident polarization. Adapted with permission from [80].

However, the additional information provided by Stokes and Mueller matrix measurements comes at the expense of increased system complexity. Data analysis is similarly computationally intensive and often requires sophisticated decomposition algorithms and careful calibration procedures. Moreover, depolarization induced by scattering in thick tissues can complicate interpretation. Despite these challenges, Stokes‐resolved SHG represents the current state‐of‐the‐art in SHG polarimetry. By providing a complete description of the polarization properties of the emitted harmonic field, these techniques offer unprecedented insights into tissue ultrastructure and molecular organization.

### 3.6 Applications of SHG Microscopy

The biomedical impact of SHG microscopy stems from its ability to visualize highly ordered, noncentrosymmetric structures without exogenous labels. Unlike histology, which provides static snapshots and requires tissue processing, SHG enables three‐dimensional, nondestructive imaging of collagen and myosin in their native environment. Consequently, the technique has evolved from a qualitative imaging modality to a quantitative tool for assessing tissue architecture, disease progression, and therapeutic response. Historically, dermatological and ophthalmic applications represented the first major successes of SHG microscopy owing to the abundance of fibrillar collagen in these tissues. Subsequent developments in polarization‐sensitive and multimodal SHG have extended its utility to musculoskeletal tissues, where quantitative assessment of ECM organization has become increasingly important. More recently, integration with machine learning and superresolution approaches has facilitated the extraction of clinically relevant biomarkers from SHG datasets, highlighting the growing translational potential of this technology.

#### 3.6.1 Skin

Skin was among the first tissues to be extensively investigated using SHG microscopy because of its highly organized dermal collagen network and the accessibility of the tissue for optical imaging. High‐resolution three‐dimensional SHG images revealed collagen fiber arrangement and orientation across the full thickness of the dermis, from the papillary layer to the reticular layer (Figure 9). Fiber density, organization, and cross‐linking can be quantified as readouts of skin aging or disease [81, 82]. SHG can be used to detect structural collagen changes in scleroderma, psoriasis, nevi, melanoma, and fungal infections [63] and has been used to track new collagen deposition during wound healing in real time [83]. In tissue engineering applications, SHG assesses how implanted dermal scaffolds integrate with native collagen, guiding scaffold design for skin regeneration.

**FIGURE 9:**
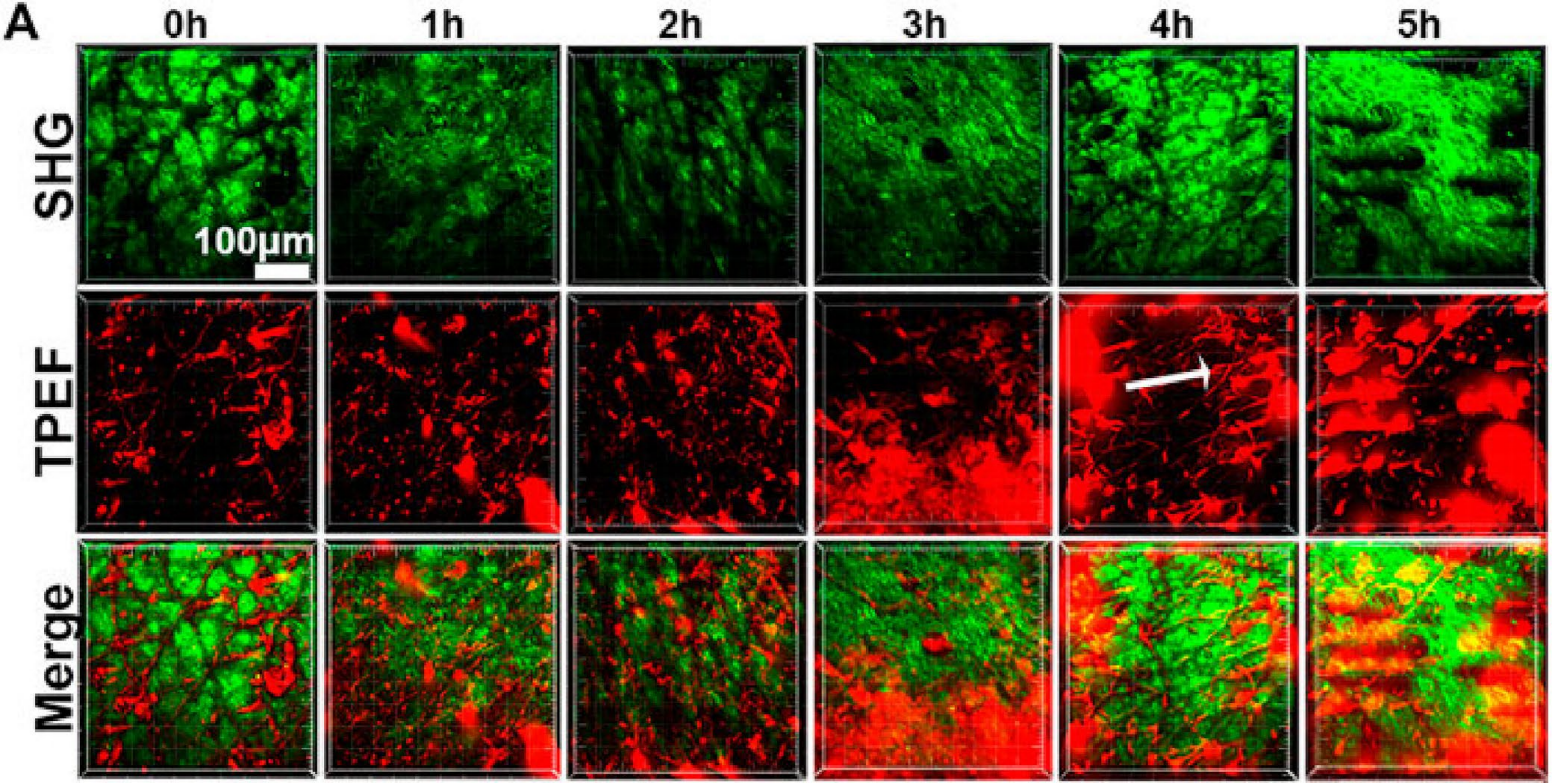
Transdermal collagen visualization by SHG‐TPEF imaging in mouse skin in vivo at 0–5 h posttreatment. Adapted from [81].

#### 3.6.2 Cornea

Following dermatological applications, the cornea emerged as an ideal model system for SHG microscopy because of its optical transparency and highly ordered lamellar collagen architecture. Conventional imaging modalities such as optical coherence tomography provide valuable morphological information. However, these modalities lack sensitivity to the collagen ultrastructure. Freund et al.'s pioneering work on collagen SHG laid the groundwork for subsequent studies of corneal organization, while later investigations demonstrated the ability of SHG to resolve stromal lamellae across the full corneal thickness [58]. SHG microscopy of the cornea revealed fibrillar collagen without staining or fixation, revealing lamellar orientation at different depths (Figure 10) [84]. Importantly, SHG has enabled quantitative characterization of diseases such as keratoconus, in which disruption of the normal orthogonal collagen arrangement represents a defining pathological feature [85]. In the context of corneal injury, SHG has been used to monitor collagen remodeling following chemical burns, providing quantitative metrics of healing that are difficult to obtain using conventional imaging methods [66, 86]. The introduction of P‐SHG and SHG‐CD has further expanded the scope of corneal imaging. These techniques permit the assessment of fibril orientation, anisotropy, and out‐of‐plane organization with submicrometric precision [67, 69].

**FIGURE 10:**
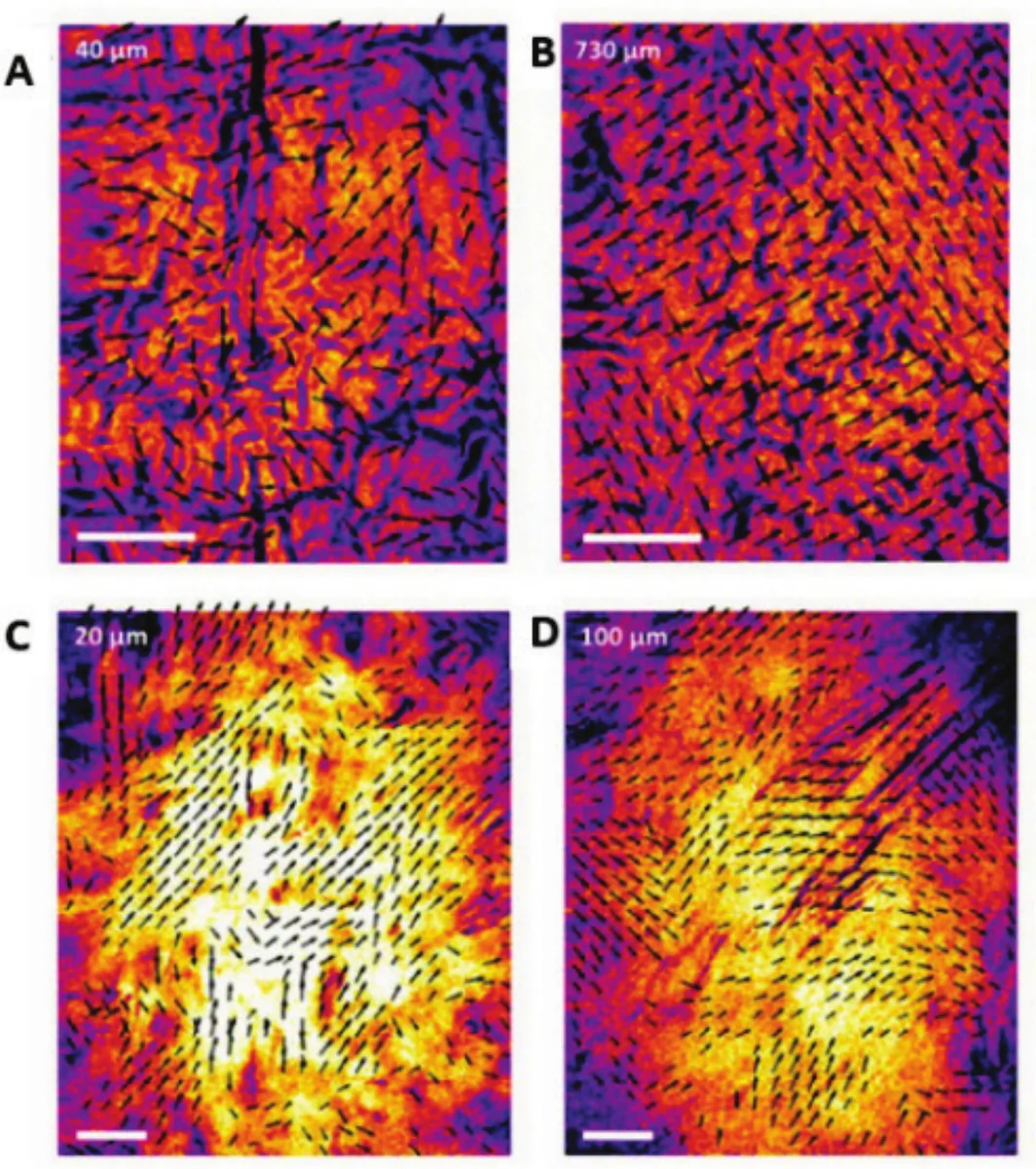
Polarization‐resolved SHG images of porcine eyeballs and rat corneas at different imaging depths, showing the fibrillar collagen orientation map. Ex vivo polarization‐resolved SHG imaging of intact porcine eyeball at (A) 40 and (B) 730 µm depth. In vivo polarization‐resolved SHG imaging from rat cornea at (C) 20 and (D) 100‐µm depth. Adapted from [84].

#### 3.6.3 Bone

Bone presents a particularly challenging imaging target because of its mineralized matrix and complex hierarchical organization. Traditional approaches for evaluating bone quality typically rely on histology, microcomputed tomography, or mechanical testing, each of which provides incomplete information regarding collagen organization. SHG microscopy addresses this gap by directly visualizing the collagen scaffold that underpins bone mechanics. Pathological bone microstructure—including changes associated with osteoporosis, type II diabetes, and osteogenesis imperfecta—is distinguishable from that of healthy bone using persistent homology analysis on SHG images [87, 88]. Polarization‐SHG distinguishes type I from type II collagen in fracture healing calluses by measuring the peptide pitch angle and anisotropy [89], and wavefront‐corrected polarization‐resolved SHG extends this approach to greater depths [90]. Time‐series SHG imaging of osteoblast cultures under cyclic strain tracks collagen secretion and fiber organization during differentiation in vitro [91, 92]. In osteogenesis imperfecta tissue, SHG captures the characteristic disorganized collagen matrix, potentially supporting clinical diagnosis [93].

#### 3.6.4 Cartilage

Cartilage imaging is among the most compelling applications of SHG microscopy. Osteoarthritis and related degenerative disorders are characterized by progressive disruption of the collagen network long before gross morphological changes become apparent. Conventional imaging modalities, including magnetic resonance imaging and radiography, primarily detect late‐stage structural alterations. In contrast, SHG is sensitive to subtle changes in collagen organization and can therefore identify early signs of tissue degeneration [94, 95, 96]. Beyond disease diagnostics, SHG has become an indispensable tool in cartilage tissue engineering. Longitudinal imaging of engineered constructs enables nondestructive evaluation of collagen deposition and maturation, facilitating optimization of scaffold design and culture conditions [97].

#### 3.6.5 Tendons and Ligaments

Collagen fibers in tendons and ligaments generate strong SHG signals, and their crimp patterns and fiber orientations can be mapped in three dimensions (Figure 11) [98]. Changes in fiber organization under mechanical loading have been characterized [99], and the advanced maximum likelihood estimation (Adv‐MLE) algorithm has been applied to recover higher spatial frequencies from SHG images, pushing the resolution closer to the diffraction limit [100, 101]. Microstructural damage not visible by conventional histology can be detected in SHG images, indicating early‐stage tissue degeneration [102]. Longitudinal studies—possibly because SHGs are nondestructive—have followed tendon development from embryonic stages through postnatal maturation, informing strategies for tendon tissue engineering [103, 104].

**FIGURE 11:**
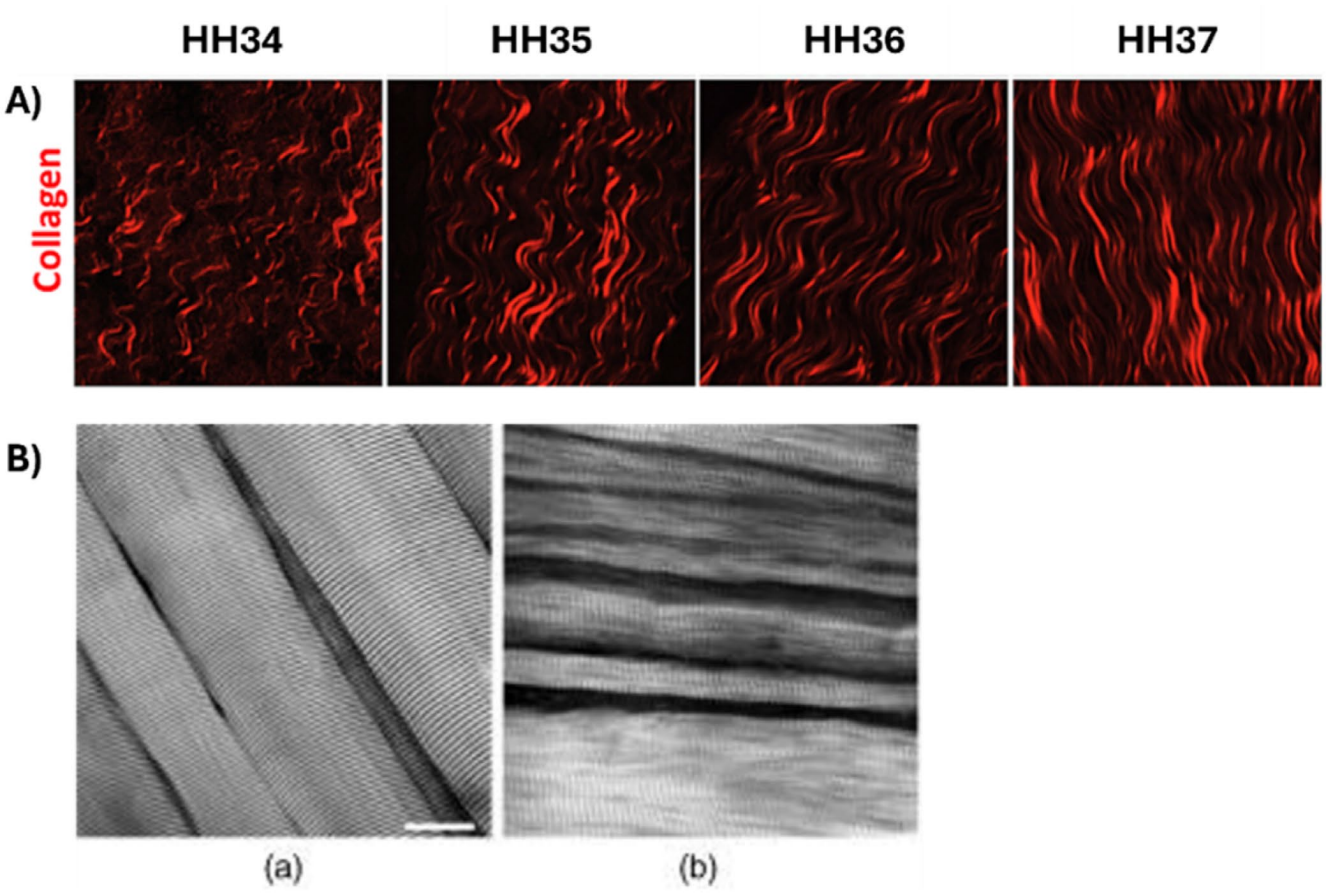
(A) SHG images of collagen fibers in embryonic chick tendons at developmental stages HH34–HH37, showing progressive fiber organization. (B) SHG imaging of (a) normal and (b) diseased skeletal muscle. Scale bar: 20 μm. Adapted from [75].

#### 3.6.6 Muscle

Unlike most SHG applications, which rely primarily on collagen, muscle imaging exploits the highly ordered arrangement of myosin filaments within sarcomeres. This distinction makes SHG uniquely suited for investigating both structural and functional aspects of contractile tissues. Myosin filaments in striated muscle generate SHG because of their ordered, non‐centrosymmetric arrangement within the sarcomere. SHG images reveal sarcomere length and the bipolar organization of myosin arrays, with signal nulls at the M‐line where filament polarity reverses. Anisotropy measurements from SHG data have been used to track conformational changes in myosin during contraction and relaxation cycles, reporting on the mechanical state of muscle in a label‐free manner [105]. Combined SHG and two‐photon autofluorescence imaging characterize elastin alongside collagen and myosin in aging muscle. Integration of SHG with two‐color stimulated emission depletion (STED) microscopy has enabled sub‐diffraction imaging of cardiomyocyte–ECM interactions, locating myosin and collagen assemblies at cell–matrix contact sites [106]. Nevertheless, dynamic muscle imaging remains challenging because motion artifacts and the relatively slow acquisition rates of conventional SHG systems can limit temporal resolution. Emerging high‐speed scanning approaches and computational reconstruction methods are expected to address these limitations and further expand the utility of SHG in functional muscle imaging.

## 4 Clinical Translation and Future Perspectives

The increasing ability of nonlinear optical microscopy to provide high‐resolution, label‐free, and depth‐resolved information from living tissues has generated considerable interest in its translation from laboratory research to clinical applications. In particular, TPF, FLIM, and SHG microscopy offer complementary information on cellular morphology, tissue metabolism, and ECM organization, respectively. The reliance of these techniques on endogenous fluorescence and SHG contrast further reduces the dependence on exogenous labels, thus making these techniques attractive for longitudinal imaging. However, successful implementation into clinical routines requires more than demonstrating high image quality in experimental specimens; the technologies must also provide clinically relevant information within an acceptable acquisition time, workflow, cost, and safety profile. Clinical translation of optical imaging generally progresses through instrument development, preclinical validation, clinical prototyping, clinical trials, regulatory approval, commercialization, and eventual integration into routine clinical practice [107].

In vivo imaging is one of the most important directions in the clinical development of nonlinear optical microscopy because it allows tissue structure and biological processes to be examined without removing the tissue from its physiological environment. In contrast to conventional histopathology, which provides a high‐resolution but static representation of an excised specimen, nonlinear microscopy can potentially provide repeated observations of the same tissue over time [108]. This capability is particularly valuable for studying disease progression, tissue regeneration, inflammation, tumor development, and response to treatment. TPF can visualize endogenous autofluorescence from molecules such as NAD(P)H and FAD, while FLIM can provide additional information related to the fluorescence lifetime and metabolic state of these endogenous fluorophores. SHG, in parallel, provides label‐free visualization of collagen and other ordered structures within the ECM. The combination of these signals can therefore provide complementary information on cellular activity and tissue architecture within the same field of view. The practical implementation of in vivo imaging, however, requires careful consideration of motion, optical access, laser exposure, imaging speed, and tissue‐induced aberrations. Furthermore, movement caused by live organisms' activities such as respiration, cardiac activity, and blood flow can introduce substantial image distortion, particularly during three‐dimensional or time‐lapse acquisition [109]. Several studies have demonstrated the feasibility of label‐free multiphoton imaging in living tissues. Multiphoton endomicroscopy has been used to visualize endogenous autofluorescence and collagen‐derived SHG signals in vivo, providing cellular and ECM information without administration of fluorescent dyes [110]. In one notable preclinical demonstration, a compact endomicroscope enabled the imaging of the mouse colon repeatedly and over a period of few days during the progression of experimentally induced inflammation, allowing changes in epithelial morphology and collagen organization to be followed longitudinally [111]. These studies illustrate an important advantage of nonlinear imaging, where pathological alterations can potentially be monitored dynamically rather than inferred from a single biopsy obtained at an isolated time point (as shown in Figure 12). In case of application in a clinical context for human samples, the most realistic initial role of in vivo nonlinear microscopy is mostly to be as an adjunctive imaging technique, rather than a replacement of established standard diagnostic procedures. The technique could provide microscopic information from suspicious regions identified using conventional clinical imaging, thereby helping clinicians determine whether a lesion warrants biopsy or further intervention.

**FIGURE 12:**
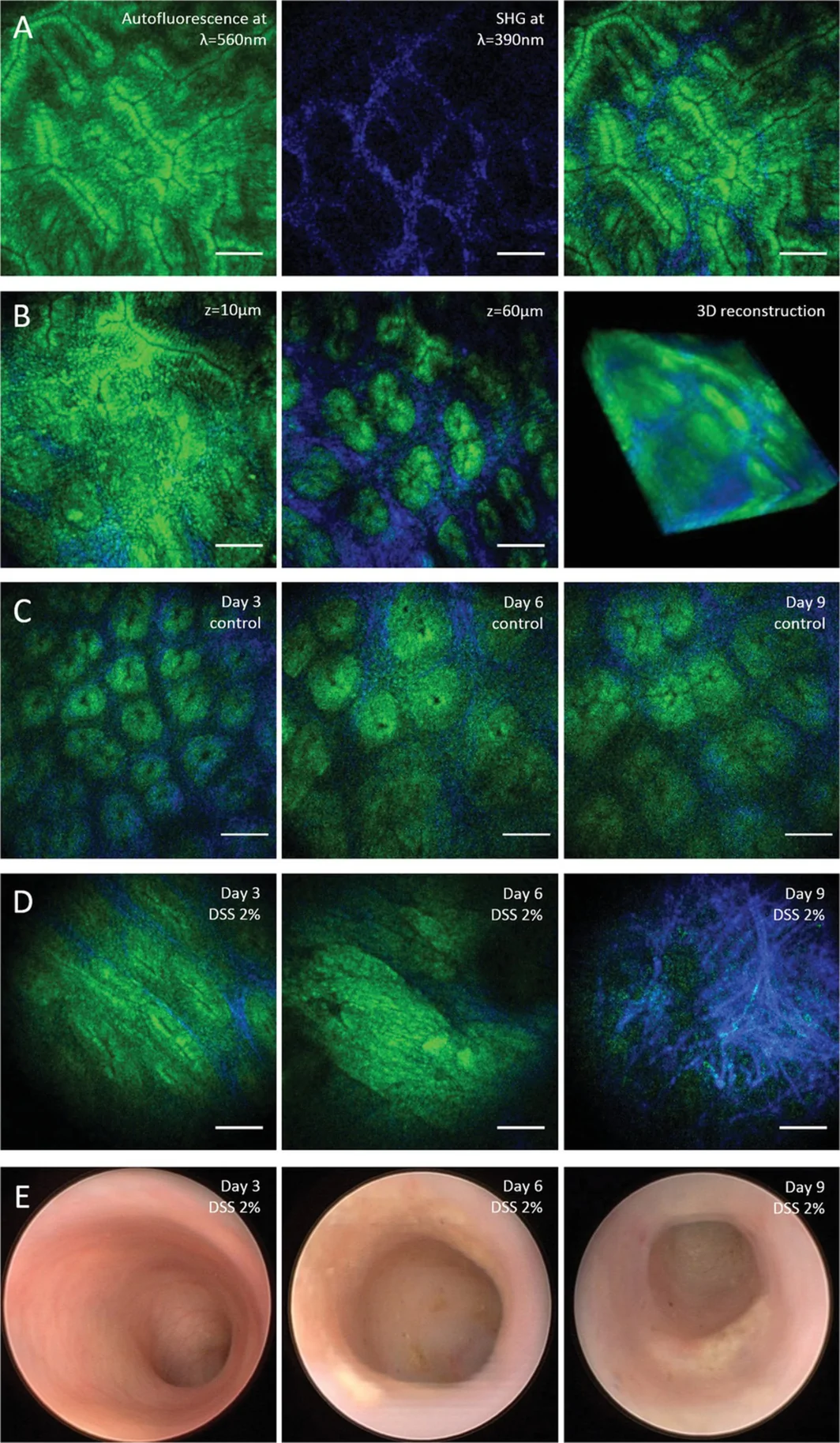
Label‐free multiphoton endomicroscopy of murine colon mucosa in vivo. (A) Two‐photon excited AF from endogenous molecules such as NADH or FAD (shown in green) and SHG from collagen‐I (shown in blue) show the epithelial crypt structure and underlying lamina of the colon mucosa. (B) Focal plane shifting by means of a fast ETL for 3D visualization of the tissue morphology. (C) Repeated minimal invasive multiphoton endomicroscopy of healthy murine colon as a control for the colitis model. (D) Changes in the tissue morphology during the time course of intestinal inflammation in a mouse model of ulcerative colitis ranging from deformations of the crypt pattern at Day 3, to massive epithelial erosion and collagen matrix formation at Day 9. Images were recorded in the same animal with minimal invasive multiphoton endomicroscopy. (E) Conventional wide‐field endoscopy as reference showing clear symptoms of acute murine DSS colitis from Days 6 to 9, and macroscopically unchanged mucosa at Day 3. Scale bars: 50 μm. Adapted with permission from [111].

To provide a comprehensive overview of the literature discussed throughout this review, Table 1 summarizes the key principles, target molecules, advantages, and applications of microscopy techniques. Furthermore, Figure 13 represents a decision tree to select the appropriate optical microscopy technique based on the required target molecules and information from tissues.

**TABLE 1:** Comparison of two‐photon microscopy techniques.

Imaging technique	Contrast mechanism	Label‐free	Typical imaging depth	Spatial resolution	Photobleaching/photodamage	Functional information provided	Major limitations
Two‐photon fluorescence (TPF) microscopy [18, 19]	Two‐photon excitation of endogenous fluorophores	Yes—endogenous auto‐fluorescence	Typically, several hundred μm in tissue [200–500 μm]; depth depends strongly on scattering, wavelength, and tissue type	Submicron to ~1 μm, depending on wavelength, NA, and system configuration	Reduced compared with single‐photon fluorescence	Cellular morphology Endogenous metabolic fluorescence Dynamic cellular processes	Limited penetration in highly scattering tissues High laser cost Relatively slow point‐scanning acquisition Complex instrumentation
Fluorescence lifetime imaging microscopy (FLIM) [35, 50]	Measurement of the fluorescence excited‐state lifetime	Yes—for endogenous fluorophores (NADH and FAD)	Conventional FLIM is depth‐limited; 2P‐FLIM can reach several hundred μm in tissue [200–500 μm]	Typically submicron–micrometer scale	Susceptible to photobleaching and phototoxicity; 2P‐FLIM reduces out‐of‐focus photodamage	Metabolic state, protein binding, FRET, pH, ion concentration, Ca^2+^ dynamics, redox state and cellular heterogeneity	Photon‐limited acquisition Long acquisition times Computationally demanding lifetime fitting Complex timing electronics
Second‐harmonic generation (SHG) microscopy [45]	Coherent nonlinear conversion of two incident photons into one photon in non‐centrosymmetric structures	Yes	Several hundred μm, depending on tissue scattering and detection geometry [200–500 μm]	Submicron to ~1 μm	Negligible intrinsic photobleaching and phototoxicity	Collagen organization Myosin arrangement Fiber architecture Tissue remodeling Extracellular matrix organization	Requires ordered/non‐centrosymmetric structures Signal depends strongly on molecular organization and polarization Specialized femtosecond lasers required
Polarization‐resolved SHG (P‐SHG) [45]	Variation of SHG intensity with excitation and/or detection polarization	Yes	Similar to conventional SHG	Comparable to SHG, generally submicron–~1 μm	Negligible intrinsic photobleaching and low phototoxicity	Quantitative information on fiber orientation Molecular anisotropy Collagen organization Nonlinear susceptibility	Requires accurate polarization control and calibration Sensitive to optical alignment and tissue heterogeneity Acquisition can be slower than conventional SHG
SHG circular dichroism (SHG‐CD) [60, 76]	Ratio between SHG signals generated under left‐ and right‐circularly polarized excitation	Yes	Similar to SHG	Comparable to SHG	Similar to SHG	Molecular chirality Macromolecular organization Molecular tilt and three‐dimensional structural arrangement	Same as P‐SHG Interpretation of chiral signals can be complex Limited clinical validation

**FIGURE 13:**
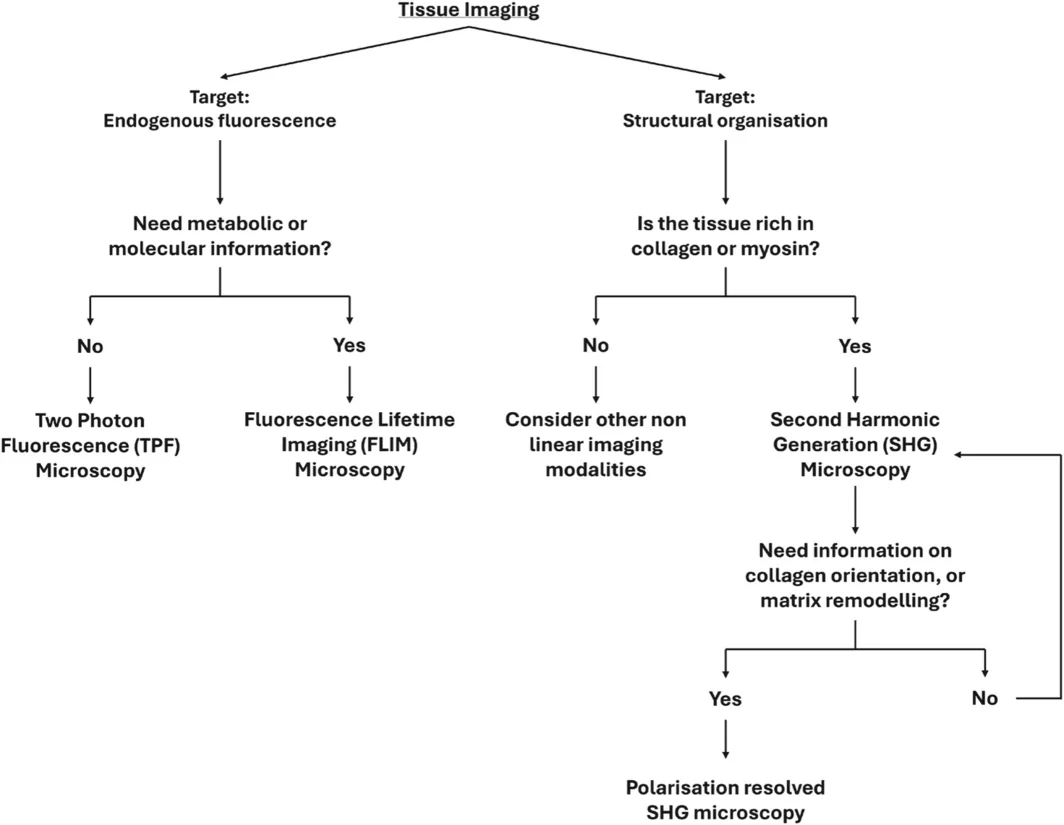
A decision tree to select the appropriate nonlinear microscopy technique for tissue imaging.

## 5 Conclusion

Over the past three decades, nonlinear optical microscopy has undergone a remarkable transformation, evolving from a collection of specialized optical techniques to a comprehensive platform for interrogating biological systems across multiple spatial and temporal scales. The development of TPM, SHG microscopy, and FLIM has progressively addressed many of the limitations associated with conventional optical imaging, including restricted penetration depth, photobleaching, inadequate functional contrast, and the dependence on exogenous labels. Together, these modalities have enabled unprecedented access to the structural, biochemical, and functional landscapes of living tissues. As instrumentation continues to improve in speed, depth, and miniaturization, TPM and SHG are well positioned for broader clinical and in vivo biomedical applications, including intraoperative assessment of tissue margins and real‐time monitoring of therapeutic responses. Despite these advances, several challenges continue to limit broader adoption and clinical translation. Imaging depth remains constrained by optical scattering and aberrations in highly turbid tissues, whereas acquisition speed and photon efficiency present significant trade‐offs in dynamic imaging applications. Furthermore, the high cost and complexity of femtosecond laser sources, together with the need for specialized expertise in instrumentation and data analysis, remain substantial barriers to widespread implementation. Importantly, the future of the field is unlikely to be defined by any single modality. Rather, the integration of TPM, SHG, FLIM, and complementary techniques into unified multimodal platforms represents a paradigm shift toward holistic tissue characterization. In conclusion, nonlinear optical microscopy has progressed from an experimental curiosity to a powerful and versatile imaging framework with broad implications for biomedicine.

## Funding

This work was supported by the ASEAN‐India Science & Technology Development Fund (AISTDF), Anusandhan National Research Foundation (ANRF/F/84/2025‐2026), Indian Council of Medical Research (D/SG‐00170/2024), and Council of Scientific and Industrial Research, Government of India, India (08/0602(23331)/2025‐EMR‐I).

## Conflicts of Interest

The authors declare no conflicts of interest.

## Data Availability

The data that support the findings of this study are available from the corresponding author upon reasonable request.
